# Identification of visual cortex cell types and species differences using single-cell RNA sequencing

**DOI:** 10.1038/s41467-022-34590-1

**Published:** 2022-11-12

**Authors:** Jia-Ru Wei, Zhao-Zhe Hao, Chuan Xu, Mengyao Huang, Lei Tang, Nana Xu, Ruifeng Liu, Yuhui Shen, Sarah A. Teichmann, Zhichao Miao, Sheng Liu

**Affiliations:** 1 State Key Laboratory of Ophthalmology, Zhongshan Ophthalmic Center, Sun Yat-sen University, Guangdong Provincial Key Laboratory of Ophthalmology and Visual Science, Guangzhou, China; 2 Wellcome Sanger Institute, Wellcome Genome Campus, Cambridge, UK; 3 Department of Physics, Cavendish Laboratory, University of Cambridge, Cambridge, UK; 4 GMU-GIBH Joint School of Life Sciences, Guangzhou Laboratory, Guangzhou Medical University, Guangzhou, China; 5 European Bioinformatics Institute, European Molecular Biology Laboratory, Wellcome Genome Campus, Cambridge, UK; 6 Guangdong Province Key Laboratory of Brain Function and Disease, Guangzhou, China

**Keywords:** Molecular neuroscience, Visual system, Cell type diversity

## Abstract

The primate neocortex exerts high cognitive ability and strong information processing capacity. Here, we establish a single-cell RNA sequencing dataset of 133,454 macaque visual cortical cells. It covers major cortical cell classes including 25 excitatory neuron types, 37 inhibitory neuron types and all glial cell types. We identified layer-specific markers including *HPCAL1* and *NXPH4*, and also identified two cell types, an *NPY*-expressing excitatory neuron type that expresses the dopamine receptor D3 gene; and a primate specific activity-dependent *OSTN* + sensory neuron type. Comparisons of our dataset with humans and mice show that the gene expression profiles differ between species in relation to genes that are implicated in the synaptic plasticity and neuromodulation of excitatory neurons. The comparisons also revealed that glutamatergic neurons may be more diverse across species than GABAergic neurons and non-neuronal cells. These findings pave the way for understanding how the primary cortex fulfills the high-cognitive functions.

## Abstract (web summary)

The laminar organization of macaque V1 cortex is similar to that seen in humans. Here the authors describe a single-cell RNA sequencing dataset of macaque V1 cortex cells.

## Introduction

The mammalian cerebral cortex is responsible for high-level cognitive functions and information processing. These functions are carried out through complicated networks of diverse cell types. Building on the morphological description of neurons by Ramón y Cajal^1^, the neuronal cell types are classified in detail based on their morphology, function, laminar distribution, projection, and developmental origin^2^. Evolution drives their structural and functional variations across species. In primates, complex sensory processing must be executed and evolution led to the development of enhanced features in many cortical areas. The primary visual cortex (V1) of macaque can be ideal to dissect the fundamental molecular and cellular drivers for such variation. The V1 in primates has evolved specialized structures such as an expanded L4^3^ (Supplementary Fig. 1a, c–e), ocular dominance columns^4^, and magnified receptive fields for the fovea^5^, raising the possibility that distinct cell types and sophisticated circuitry can support the complex visual function. The laminar organization of macaque V1 is similar to that seen in humans, as revealed by both the distribution of Nissl staining and the cell-level molecular patterns^6^. The macaque V1 is a great model to study molecular underpinnings of the evolution of the brains across species including the cellular and molecular shifts towards higher-order primates such as humans.

Recent advances in high-throughput sequencing technologies added new modalities to the classification of neuronal cell types^7–17^. Profiling of the transcriptome, chromatin accessibility, and epigenome at the single-cell level provides a means to understand the cellular and molecular mechanisms underlying both normal brain function and disease^18–27^. For example, a comprehensive transcriptomic atlas of the isocortex and visual system has been established for rodents and later in primates^17,28–32^. Retrograde labeling^33,34^, gene manipulation^35^, and experience-dependent experiments^36,37^ in rodents further integrate the functional and transcriptomic annotations to the atlas and set the framework for the primate atlas^22,38^.

Comparative studies of developing and mature nervous systems across species revealed both conservation and divergence of gene-expression patterns across species, establishing the framework for elucidating the augmented complexities in the primate neocortex^14,15,19,22,29,39,40^. Yet, the driver genes that maintain the diversity of the neuronal transcriptome types, and ultimately, the physiological and morphological properties^7^ of the adult primate neurons, have not been investigated in detail.

The macaque V1, which is under extensive study for vision cognition^41,42^, can be ideal to address this topic. V1 is the largest visual cortical area that plays a critical role in visual information processing^43,44^. Visual experience drives the maturation and maintains the established function of the V1^36,37^. The visual experience still drives network plasticity, to some extent, after the maturation in primates^45^, further suggesting the intensive interplay between external inputs and the intrinsic gene-expression regulation in adulthood. Deciphering the underlying cellular and molecular basis may facilitate our understanding of how the neural network is maintained in adulthood, and may facilitate our understanding of neurodegenerative diseases such as Alzheimer’s and Huntington’s^25^.

Here, we extracted the whole-cell bodies from the macaque V1 and established a large-scale dataset to uncover novel cell types and critical neuronal features at the single-cell level. We obtain healthy cell bodies from 133,454 V1 cells and depict the whole-cell transcriptomic landscape of primate V1. Detailed comparisons of our dataset with human and mouse single-cell transcriptomics datasets reveal novel excitatory cells and cell populations that involve the primate-specific activity regulatory mechanism. The divergence of the excitatory neurons between primates and rodents lies in gene-expression modules associated with experience- and activity-regulated synaptic plasticity.

## Results

### Molecular atlas of macaque V1

**Fig. 1:**
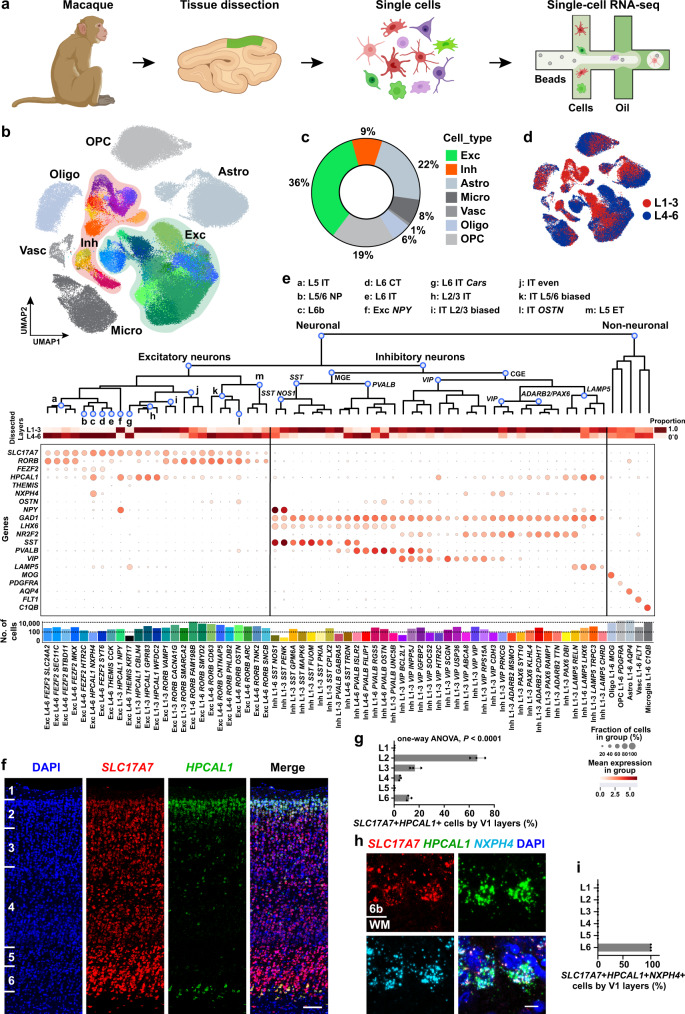
Cell type taxonomy in the cynomolgus monkey primary visual cortex (V1). **a** Schematic illustration of single-cell transcriptome profiling of the macaque primary visual cortex (V1). The macaque V1 used for single-cell isolation and staining experiments is visualized in green. Drawings of macaque and single cells were created with BioRender.com. **b** Uniform Manifold Approximation and Projection (UMAP) visualization of the 133,454 cells of macaque V1. Exc excitatory neurons, Inh inhibitory neurons, Astro astrocytes, Oligo oligodendrocytes, OPC oligodendrocyte progenitor cells, Micro microglia, Vasc vascular cells. **c** Quantification of the percentage of different major cell types revealed by scRNA-seq datasets in macaque V1. **d** UMAP visualization colored by the enrichment dissection, cells collected from the tissue covering all layers are not shown. **e** The taxonomy of the 62 neuronal cell types (including 25 excitatory and 37 inhibitory types) and 5 non-neuronal cell types from the macaque V1. Branches are labeled with major cell populations.The dot-plot shows the mean expression level of selected genes. Cell types are labeled with layer distribution and their marker genes. Exc excitatory neurons. Inh inhibitory neurons. Astro astrocytes, Oligo oligodendrocytes, OPC oligodendrocyte progenitor cells, Vasc vascular cells. IT intratelencephalic, ET extratelencephalic, NP near-projecting, CT corticothalamic. **f** RNAscope mFISH confirms that *HPCAL1* (green) is mainly expressed in a subset of *SLC17A7* (red) glutamatergic cells in L2/3 and L6b. Blue, DAPI. Scale bar, 100 μm. **g** Quantifications of *SLC17A7*+*HPCAL1*+ cells across layers of macaque V1. Data are represented as mean ± s.d. and dots show the data points for individual macaques. The differences between groups were significant, as determined by one-way ANOVA. *n* = 3 macaques. **h** RNAscope mFISH for mRNA of *SLC17A7* (red), *HPCAL1* (green) and *NXPH4* (light blue) are co-localized in L6b. Blue, DAPI. 6b, layer 6b; WM, white matter. Scale bar, 10 μm. **i** Counts of *SLC17A7*+*HPCAL1*+*NXPH4*+ cells. Data are represented as mean ± s.d. and dots represent biological replicates. *n* = 3 macaques. Source data are provided as a Source Data file.

Using a specifically developed scRNA-seq pipeline (see Methods) tailored for improving neuronal survival, we established a dataset of whole-cell transcriptomes from the cynomolgus monkeys (*Macaca fascicularis*) V1 (Fig. 1a; Supplementary Fig. 1). These cells came from live tissues and a number of them had morphologies characteristic of neurons (Supplementary Fig. 1a, b). The laminar organization and expanded layer (L) 4 of cynomolgus monkey V1 are similar to humans^46^, as revealed by the distribution of Nissl staining and Golgi staining (Supplementary Fig. 1a, c–e). We acquired 133,454 high-quality single-cell transcriptomes with a median of 3293 unique molecular identifiers (UMIs) and 1904 genes per cell (Supplementary Fig. 1f–i).

The cells are classified by three hierarchically-related levels: classes, subclasses and cell types. Based on the expression of canonical markers, cells were classified into seven classes: four classes of glial cells (72,490 cells, 54%), vascular cells (1107 cells, 1%), excitatory neurons (47,760 cells, 36%), and inhibitory neurons (12,097 cells, 9%) (Fig. 1b, c). Their laminar distribution varies (Fig. 1d). The neuronal populations consist of 80% excitatory and 20% inhibitory neurons (Fig. 1c), in keeping with the expected ratio in the primate cortex^39,47^ and slightly different from that of rodents (85% vs. 15%)^48–51^. The excitatory and inhibitory neuronal classes are divided into 13 and five subclasses, respectively. Subclasses may be subdivided, yielding 25 excitatory and 37 inhibitory neuron cell types. These neuron cell types, together with the five non-neuronal cell types, establish a taxonomy of 67 distinct cell types (Fig. 1e). The neuron cell types are named consistently^52^, that is, each cell type is named after its class, followed by the laminar distribution and two characteristic marker genes. The first marker gene can be shared among several cell types, reflecting its laminar distribution (for excitatory neurons) or subclasses (for inhibitory neurons). The second marker gene is distinct for each cell type (Fig. 1e). The non-neuronal cell types are named following the same rule, with one marker gene already being sufficient to characterize each cell type. The detailed illustrations for the relationship among classes, subclasses, cell types, and marker genes are summarized in Fig. 1e.

### Cellular and molecular profiling of excitatory neurons

**Fig. 2:**
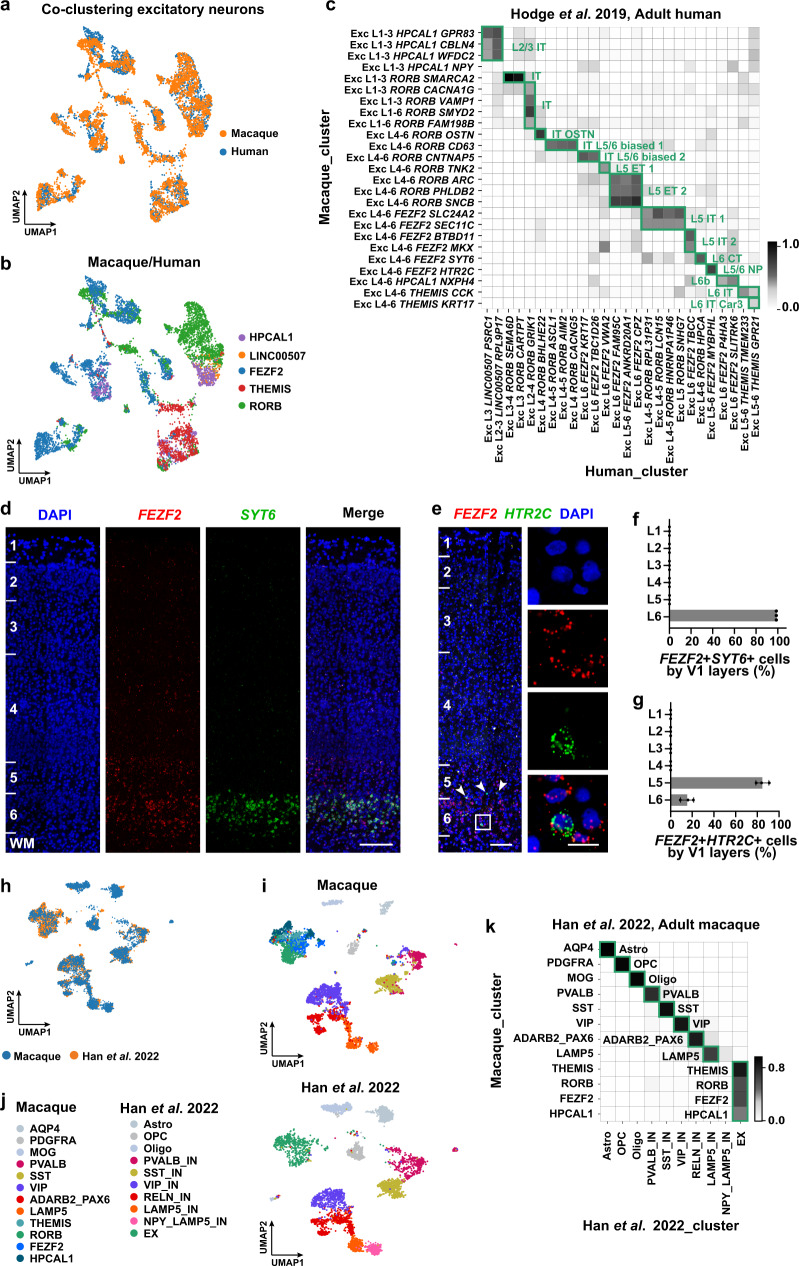
Cell types of macaque V1 are aligned with well-established human and macaque datasets. **a** UMAP visualization of the integration of the combination of macaque (downsampled to *n* = 4728 neurons) and human (*n* = 3158 neurons) excitatory neurons using Harmony. **b** UMAP visualization of the macaque and human excitatory neurons clusters after alignment. **c** Cell-type homology of macaque and human excitatory neurons, estimated by shared cluster membership between the two species. Gray shade corresponds to the proportion of co-clustering between macaque cells and human nuclei. Rows show macaque cell types and columns show human cell types. **d** RNAscope mFISH shows the co-localization of *FEZF2* (red) and *SYT6* (green) in L6 of macaque V1. Scale bar, 200 μm. **e** RNAscope mFISH confirms that *HTR2C* (green) is mainly expressed in a subset of *FEZF2* (red) glutamatergic cells in L5/6. Blue, DAPI. Co-labeled cells in L5 are indicated by white arrowheads. Scale bars, low magnification, 100 μm; high magnification, 20 μm. **f** The quantification of *FEZF2*+*SYT6*+ across layers of macaque V1. Data are mean ± s.d. and individual replicates are shown as dots. *n* = 3 macaques. **g** The quantifications of cell distribution across layers of macaque V1. Count of *FEZF2* + *HTR2C* + cells. Data are mean ± s.d. and dots show the data points for individual macaques. *n* = 3 macaques. **h** UMAP visualization of the integration between two macaque datasets (*n* = 6023 cells from our dataset and *n* = 4823 cells from Han et al. snRNA-seq dataset of neocortex). **i** UMAP visualization of macaque V1 cell annotations using the same UMAP coordinates in (**h**). **j** UMAP visualization of macaque cell dataset from Han et al. using the same UMAP coordinates in (**h**). Astro astrocytes, Oligo oligodendrocytes, OPC oligodendrocyte progenitor cells, EX excitatory neurons, IN inhibitory neurons. **k** Cell type homologies between our dataset and the Han dataset, predicted based on the shared cluster membership. Rows show our macaque populations, and columns show the Han dataset. Source data are provided as a Source Data file.

**Fig. 3:**
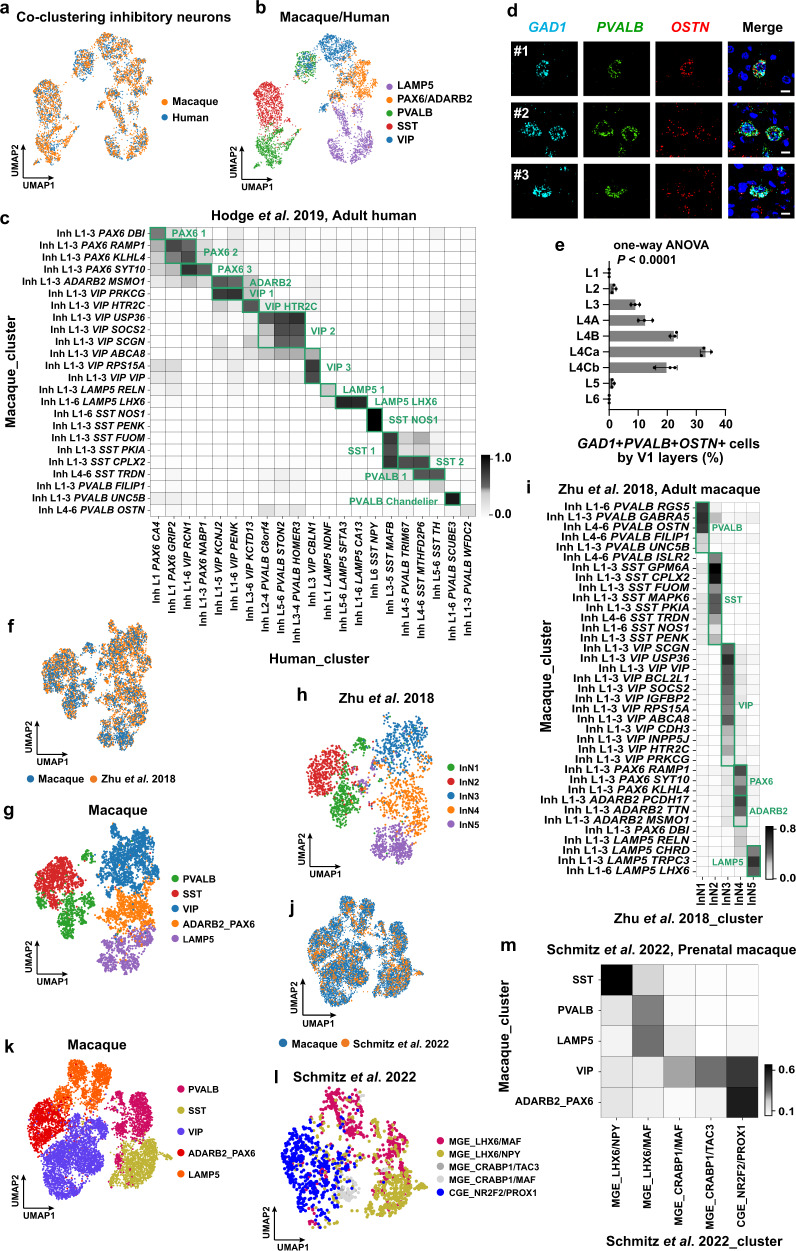
Inhibitory cell types of macaque V1 are aligned with well-established human and macaque datasets. **a** UMAP visualization of the integration of the combination of macaque (downsampled to *n* = 2473 neurons) and human (*n* = 2325 neurons) inhibitory neurons using Harmony. **b** UMAP visualization of the Harmony integrated macaque and human inhibitory neurons, colored by their subclasses. **c** Cell-type homology of macaque and human inhibitory neurons, estimated by shared cluster membership between the two species. Gray shade corresponds to the proportion of co-clustering between macaque cells and human nuclei. **d** RNAscope mFISH showing co-expression of *OSTN* (red) with inhibitory neurons *GAD1* (light blue) and *PVALB* (green) gene. Scale bar, 10 μm. **e** Percentage of triple-positive cells across layers. Data are represented as mean ± s.d. and dots show the data points for individual macaques. The differences between groups were significant, as determined by one-way ANOVA. *n* = 3 macaques. **f** UMAP visualization of the integration between the two macaque datasets (*n* = 4903 cells from our dataset, and *n* = 2500 cells from Zhu et al.). **g** UMAP visualization of macaque V1 inhibitory neuron annotations using the same UMAP coordinates in (**f**). **h** UMAP visualization of inhibitory macaque neuron annotations from Zhu et al. using the same UMAP coordinates in (**f**). InN inhibitory neurons. **i** Cell type homologies between our dataset and Zhu dataset, predicted based on the shared cluster membership. **j** UMAP visualization of the integration between two macaque datasets (*n* = 8315 cells from our dataset and macaque, and *n* = 1619 cells from Schmitz et al.). **k** UMAP visualization of macaque V1 inhibitory neuron annotations using the same UMAP coordinates in (**j**). **l** UMAP visualization of inhibitory neuron annotations from Schmitz et al. using the same UMAP coordinates in (**j**). Abbreviations in Schmitz UMAP: MGE medial ganglionic eminences, CGE caudal ganglionic eminences. **m** Cell-type homologies between the inhibitory neurons in our dataset and Schmitz dataset, predicted based on the shared cluster membership. Source data are provided as a Source Data file.

**Fig. 4:**
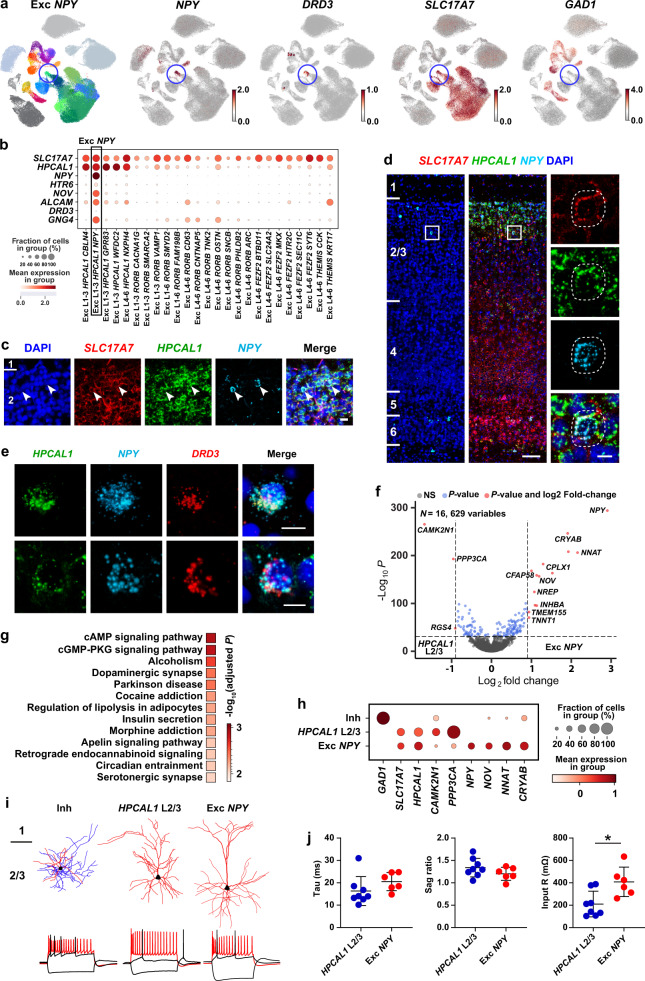
Exc *NPY* is a novel excitatory neuron type. **a** UMAP visualization of the Exc *NPY* (Exc L1-3 *HPCAL1 NPY*) neurons, blue circles indicate the Exc *NPY* cells. The Exc *NPY* neurons express markers including *NPY*, *DRD3* and the excitatory neuron marker *SLC17A7*, but not the inhibitory neuron marker *GAD1*. **b** Dotplot shows the distribution of Exc *NPY* cell marker gene-expression across all excitatory cell types. c-d) RNAscope mFISH showing representative *SLC17A7*+*HPCAL1*+*NPY*+ cells. Red, *SLC17A7*; Green, *HPCAL1*; Light blue, *NPY*. Labeled cells are indicated by white arrowheads in (**c**). Scale bar, 20 μm in (**c**); low magnification for 100 μm and high magnification for 10 μm in (**d**). *n* = 3 macaques. **e** RNAscope mFISH showing representative *HPCAL1*+*NPY*+*DRD3*+ cells. Green, *HPCAL1*; Light blue, *NPY*; Red, *DRD3*. Scale bar, 10 μm. *n* = 3 macaques. **f** Volcano plot shows the differential expression genes (DEGs) between the *HPCAL1* L2/3 and Exc *NPY* subclasses. NS, not significant; blue dots, *P* value <10e−32; red dots, *P* value <10e−32 and log_2_ fold-change > 0.9. *P*-values were determined by a two-part generalized linear model implemented by MAST. **g** KEGG enrichment analysis reveals the Exc *NPY* upregulated DEGs (*n* = 200) are overlapped with Alcoholism, Dopaminergic synapse, and Addiction signaling pathways. *P-*value for each term was determined by Fisher’s exact test implemented in (**g**). Profiler with FDR-adjust for multiple comparisons, indicated as the color-coded squares. **h** Dotplot highlighting transcriptional differences among Inh, *HPCAL1* 2/3 and Exc *NPY*. Inh, inhibitory neurons; Exc, excitatory neurons. **i** Top panel, representative morphologies for each cell type. For inhibitory neurons, dendrites are shown in red colors, axons are shown in blue colors. For excitatory neurons, only dendrites are shown. Black dots mark soma locations. Bottom panel, spiking responses to step currents for the three morphologically defined cell types. **j** Plots show electrophysiological properties measured in *HPCAL1* L2/3 and Exc *NPY* types. Input R, input resistance. Circles represent single cells. Bars represent mean ± s.d. The significance of differences in input R was determined using two-tailed Mann-Whitney test (*P < 0.05; *P* value for Tau is 0.081; for Sag ratio, 0.282; for Input R, 0.02). The *n* = 8 for *HPCAL1* L2/3; *n* = 6 for Exc *NPY*.

Diverse types of excitatory neurons are dedicated to mediating multiple processing streams and output channels in the cerebral cortex^53^. In contrast to inhibitory neurons, excitatory neurons share the same developmental origin^54,55^. They are often defined by their marker expression, laminar distributions and axon projections^31,56,57^. We leveraged four complementary approaches to classify the macaque excitatory cells: (1) their laminar distributions from our layer-enriched dissection protocol (Fig. 1d; Supplementary Fig. 1a), (2) expression of canonical/novel laminar markers (Fig. 1e–i), (3) their transcriptomic similarities (Fig. 2), and (4) machine learning-based cell type annotation prediction^58^ using mouse^28,31^ and human^56^ annotations (see Methods). Building on these approaches, we identified five larminar markers for the excitatory neurons and categorized the macaque excitatory neurons into 13 subclasses (Fig. 1e; Supplementary Fig. 2a) and 25 cell types (Fig. 1b, e; Supplementary Fig. 2b, c).

The five laminar markers for the excitatory neurons include: *HPCAL1* (expressed in L2/3 and L6b), *RORB* (L3-5), *FEZF2* (L4-6), *THEMIS* (L4-6), and *NXPH4* (L6b). *HPCAL1*, a calcium-dependent regulator for neuronal signaling^59^, shares a similar laminar expression pattern with the long intergenic non-protein coding gene *LINC00507* in the human upper layers^56^, while being additionally expressed in L6b. *NXPH4* (Neurexophilin-4), which is only enriched in L6b, distinguishes L2/3 and L6b *HPCAL1*+ cells. Using RNAscope multiplex fluorescence in situ hybridization (mFISH), we validated the laminar distribution of *HPCAL1*, *RORB*, *NXPH4* and *FEZF2* (Figs. 1f–i, 2d–g; Supplementary Fig. 2d–f). The layer distributions of the expression pattern for *RORB* and *FEZF2* are consistent with the previous publications in adult humans and mice^6,23,31,39,46,56^. We further checked the expression pattern of *HPCAL1* of the human cortex in the spatial transcriptome dataset (Human Cerebral Cortex 1, 10x Genomics) (Supplementary Fig. 3). The *HPCAL1* expression was enhanced in L2 (Supplementary Fig. 3d), consistent with its spatial topography of gene expression in the dorsolateral prefrontal cortex (dlPFC) using the same platform^60^.

The *HPCAL1*+ excitatory neurons were divided into three subclasses: *NXPH4*- L2/3 intratelencephalic (L2/3 IT), L2/3 *NXPH4*− *NPY*+ (Exc *NPY*) and *NXPH4*+ L6b subclass (Fig. 1e; Supplementary Fig. 2a, b). We further divided each subclass into transcriptomic cell types, which are defined as the minimal cell clusters with distinct gene-expression patterns^56^. Some of these subclasses contain several transcriptomic cell types while some contain only one transcriptomic cell type. For the three subclasses of *HPCAL1*+ excitatory neurons, the L2/3 IT subclass contains three transcriptomic cell types: Exc L1-3 *HPCAL1 CBLN4*, Exc L1-3 *HPCAL1 GPR83*, and Exc L1-3 *HPCAL1 WFDC2*; while the Exc *NPY* subclass contains only one cell type Exc L1-3 *HPCAL1 NPY*, and L6b subclass contains only the Exc L4-6 *HPCAL1 NXPH4*. The *RORB*+ neurons cover four subclasses of IT neurons: L2/3-enriched (IT L2/3 biased, *n* = 2909 from layers 1–3 and 210 from layers 4–6), L5/6-enriched (IT L5/6 biased, 404/4727), evenly-distributed (IT even, 5241/6920), and IT-OSTN subclass that expresses the anthropoid primate-specific neural activity-induced *OSTN* gene^61^ (Fig. 1e; Supplementary Fig. 2a, b). L5 extratelencephalic (ET) cells are also *RORB*+ neurons with a weak expression of *FEZF2* (Fig. 1e; Supplementary Fig. 2a, b). The rest of the *FEZF2*+ neurons cluster into L5 IT, L5/6 near-projecting type (NP), and L6 corticothalamic (CT) subclasses (Fig. 1e; Supplementary Fig. 2a, b). RNAscope mFISH confirmed enrichment of *FEZF2* and *SYT6* in L6, as well as *FEZF2* and *HTR2C* in L5/6, respectively (Fig. 2d–g). *THEMIS*+ neurons contain the L6 IT and L6 IT *Car3*^31,39,56^ subclasses (Fig. 1e; Supplementary Fig. 2a–c, g, h). The cell types that belong to each subclass are illustrated in Fig. 1e.

### Taxonomy of inhibitory neurons in macaque V1

The Uniform Manifold Approximation and Projection (UMAP) distribution of the macaque inhibitory neurons are split into several branches, reflecting their developmental origins^15^, including the *NR2F2*+ branch from the caudal ganglionic eminence (CGE) and the *LHX6*+ branch from the medial ganglionic eminence (MGE) (Supplementary Fig. 4a). Similar to humans^38,56^ and mice^31^, the CGE-originated branch can be further divided into the *VIP*+, *ADARB2*+/*PAX6*+ and *LAMP5*+ subclasses (Supplementary Fig. 4b) while the MGE-originated branch includes the *PVALB*+ and *SST*+ subclasses (Supplementary Fig. 4c). Notably, *HTR3A*, which marks the CGE-derived inhibitory neurons in mice^62^, is only sparsely expressed in macaques (Supplementary Fig. 4b, c), similar to its expression pattern in humans^38,56^.

These inhibitory neurons were further divided within each subclass, yielding 37 neuron types (Fig. 1e; Supplementary Fig. 4a–c). Layer-enriched dissection provides additional information for the distribution differences between MGE- and CGE-originated cell types (Fig. 1d; Supplementary Fig. 4a–c). The CGE-originated cells, including 12 *VIP*+ and seven *ADARB2*+/*PAX6*+ cell types, are mostly distributed in the upper layers (Fig. 1e; Supplementary Fig. 4a, b), consistent with those in humans and mice^31,56^. These cell types mostly express *NR2F2*, which is enriched in the upper layers and match our multilayer scRNA-seq predictions (Supplementary Fig. 4b, d). By contrast, the MGE-originated cell types are more broadly distributed (Supplementary Fig. 4e). For the *PVALB* subclass: the *PVALB RGS5* cells are distributed across all layers; the *PVALB GABRA5* and the chandelier cell-like cells *PVALB UNC5B* are distributed in upper layers; the *PVALB OSTN*, *PVALB ISLR2*, and *PVALB FILIP1* cells are distributed in deep layers (Fig. 1e; Supplementary Fig. 4a, c). The RNAscope mFISH staining further validated the chandelier cell-like cells in primates (Supplementary Fig. 4f). The *SST* subclass is also not spatially restricted. The *SST NOS1* cells are distributed across all layers; the *SST TRDN* cells are mainly distributed at the deep layers; the rest of SST cells are distributed at the upper layers (Fig. 1e; Supplementary Fig. 4a, c). The macaque Inh L1-6 *SST NOS1* cell type is demonstrated by expression of marker genes *NOS1*, *NPY* and *CHODL* (Supplementary Fig. 4c). The RNAscope mFISH and immunofluorescent staining further validated their existence and distribution in primate (Supplementary Fig. 4g, h).

The MGE and CGE branches cover all the inhibitory neuron types except for the Inh L1-6 *LAMP5 LHX6* cells. This cell type expresses both the CGE marker *ADARB2*, and the MGE marker *LHX6*^63^ (Supplementary Fig. 5a). This cell type is abundant and comprises 3.9% (467/12,097) of interneurons in macaques, similar to humans and marmosets but different from mice^38,56^. Also, similar to the human and marmoset, the distribution of macaque *LAMP5 LHX6* cells is not restricted to deep layers, but is further expanded to upper layers. This expanded distribution is validated by both their distribution in layer-enriched samples (Fig. 1d) and RNAscope mFISH (Supplementary Fig. 5b–d).

### Age effect on the cell composition and gene-expression pattern

We next sought to evaluate the effect of age on the macaque V1 cells. For the majority of the cell populations, the proportions within each sample are comparable between the young adults (4–6 years old, *n* = 5 animals and 16 samples) and middle-aged adults (13–15 years old, *n* = 3 animals and 11 samples). Statistical comparisons for inhibitory, excitatory and non-neuronal subclasses reveal that the cell proportions across samples between the two age groups do not differ significantly, except for the *RORB*+, *FEZF2*+ and *VIP*+ populations (Supplementary Fig. 6a).

To systematically compare the gene-expression profiles between the two age groups, we performed differential gene-expression (DGE) analysis for each inhibitory subclass and the *HPCAL1*+, *RORB*+, *FEZF2*+, and *THEMIS*+ cell populations of excitatory neurons. In the PVALB subclass (Supplementary Fig. 6c), 2830 genes are significantly altered between the two age groups (FDR < 0.05, out of 18,297 genes, see details in Methods), while none of these genes have more than twofold changes between the age groups. Similarly, a limited number of genes display more than twofold changes (Log_2_FC > 1) in other cell populations (Supplementary Fig. 6b, d).

We also compared the gene-expression patterns between the two age groups, focusing on the neuron differentiation-related genes (*n* = 2006, see Methods). The gene-expression profiles for young and middle-aged adults are highly correlated (Supplementary Fig. 6e), with a limited number of genes showing more than twofold expression differences. These results are consistent with the DGE analysis and further support a conserved expression pattern from young to middle-aged adults. Despite the high similarities between the two age stages, differences have been reported^14^. Indeed, there exist genes that are slightly shifted (> 0.5-fold-change, examples are shown in Supplementary Fig. 6f).

### Homologous cell types across species

To systematically compare the cellular transcriptomic architectures across species, we integrated our scRNA-seq datasets from the macaque V1 with the cell transcriptomes of the human cortical areas^31,56^ (Figs. 2a–c, 3a–c; Supplementary Fig. 7). The homologous cell types were identified for those with a high proportion of shared cluster members. All the subclasses are one-to-one aligned across species. For the glutamatergic subtypes matched to the human cortical area (Fig. 2a–c), some of them are matched to established cell types (Fig. 2c). For example, the only cell type in the L6 IT Car3 subclass, the Exc L4-6 *THEMIS KRT17*, is equivalent to mouse L6 IT VISp Car3 cells^31^, expressesing its marker genes *NR2F2* and *OPRK1* (Supplementary Fig. 2h). The macaque Exc L4-6 *THEMIS KRT17* cell type aligns with the human Exc L5-6 *THEMIS GPR21* cell type that belongs to the L5/6 IT Car3 cells^31,56^.

Similarly, major GABAergic cell types are aligned. (Fig. 3a–c). Several well-established GABAergic cell types are one-to-one matched between species. For example, the macaque Inh L1-6 *SST NOS1* cell type is homologous to human *SST NPY*, which is the equivalent to the mouse *Sst-Chodl Nos1*+ long-range projecting interneurons^31,56^ (Fig. 3c). *Sst Npy* was mainly restricted to L5/6 in mice^31,56,64^, but was found in L1 to L6 in macaques, consistent with the human cortex^39^. The macaque Inh L1-3 *PVALB UNC5B* cell type is homologous to the human Inh *PVALB SCUBE3* chandelier cells^31,56,64^ (Fig. 3c).

For non-neuronal cells, we found all major cell types described in humans^56^, including: astrocytes (Aquaporin-4, *AQP4*+, *n* = 29,540 cells), oligodendrocyte progenitor cells (platelet-derived growth factor receptor A, *PDGFRA*+, *n* = 24,977 cells), oligodendrocytes (Myelin Oligodendrocyte Glycoprotein, *MOG*+, *n* = 7668 cells), microglia (Complement C1q B Chain, *C1QB*+, *n* = 10,305 cells) and vascular cells (Fms Related Receptor Tyrosine Kinase 1, *FLT1*+, *n* = 1107 cells) (Supplementary Fig. 7a, b). Similar to the inhibitory and excitatory neurons, the integration shows highly preserved cell identities of all these cell classes between the macaque and human cortex (Supplementary Fig. 7c, d).

Consistently, alignments with additional datasets of mature^14,16^ and developing^15^ macaques yields similar results (Figs. 2h–k, 3f–m). Using a mature adult cynomolgus macaque dataset^16^, the two datasets are one-to-one aligned for both the non-neuronal classes (including astrocytes, OPCs, and oligodendrocytes) and the inhibitory neuron subclasses (PVALB, SST, VIP, LAMP5, and RELN/ADARB2-PAX6 subclasses). All of the excitatory neuron types in our dataset also map to the corresponding excitatory clusters (Fig. 2k). For the alignment with an adult Rhesus Macaque (*Macaca Mulatta*) dataset^14^, each inhibitory neuron subclass aligns with a unique cluster (Fig. 3f–i).

In addition, the cell type alignment with developing macaque inhibitory neurons^15^ is consistent with their spatial origins during development: the SST subclass aligns with the MGE_LHX6/NPY class; the PVALB and LAMP5 subclasses align with the MGE_LHX6/MAF class; the CGE_NR2F2/PROX1 class aligns with the rest of the inhibitory subclasses (VIP and ADARB2/PAX6) (Fig. 3j–m). A primate-specific class MGE CRABP1/TAC3 in the developing macaque, which gives rise to a distinct *VIP*+ class in the striatum^38^, aligns with the VIP cells in our dataset.

### Novel excitatory neuron types revealed in primates

Here, we report a novel excitatory *NPY*+ cell type, Exc *NPY* (Exc L1-3 *HPCAL1 NPY*, the only cell type under the *HPCAL1*+ Exc *NPY* subclass, Fig. 4a, b; Supplementary Fig. 8a). This cell type co-expresses *NPY* and canonical glutamatergic neuronal marker *SLC17A7*, but not the GABAergic neuronal marker *GAD1* (*n* = 417 cells, Fig. 4a, b; Supplementary Fig. 8b, c). Using RNAscope mFISH staining of *SLC17A7*, *HPCAL1* and *NPY*, we validate its presence in the macaque V1, with cell distributions clearly restricted to L2/3 (Fig. 4c, d; Supplementary Fig. 8d, e). Neuropeptide Y (NPY) is one of the most abundant and widely distributed neuropeptides in the central nervous system^65,66^. It is involved in a broad range of physiological functions, such as food intake^67^, circadian rhythms^68^ and memory^69^. Cortical *NPY*+ neurons are only known as GABAergic^70^. One of the Exc *NPY* cell marker genes is *DRD3* (Fig. 4a, b, e; Supplementary Fig. 8f), which encodes a dopamine receptor subtype that is involved in addiction^71–73^, further supporting the distinct function of the Exc *NPY* cells.

UMAP visualization shows that the Exc *NPY* cells are close to the excitatory *HPCAL1*+ L2/3 IT subclass (Fig. 4a; Supplementary Fig. 8a). Both of them express *HPCAL1* and *SLC17A7* (Fig. 4b). However, Exc *NPY* is transcriptomically distinct from the L2/3 IT cells. Multiple important transcriptional factors are differentially-expressed between the Exc *NPY* and L2/3 IT, including *NNAT* and *NOV* (Fig. 4f; Supplementary Data 1). *NNAT* (Neuronatin) regulates ion channels during development and maintains nervous system structure during adulthood^74^, and *NOV* encodes extracellular matrix-associated signaling proteins^75,76^. Additional differentially-expressed genes are shown in the volcano plot (Fig. 4f). The KEGG enrichment analysis of Exc *NPY* upregulated genes highlights its biological relevance with reward and addiction (Fig. 4g; Supplementary Data 2), further suggesting the role of Exc *NPY* in neuromodulation. Interestingly, the spatial transcriptomic dataset of the human cortex contains wells that express *SLC17A7*, *HPCAL1* and *NPY* (Supplementary Fig. 8c), indicating the Exc *NPY* may also exist in humans. However, the co-expression revealed by this approach may result from an aggregation of cells from multiple cell types. Further validations using brain tissues from different species (in particular, humans) are necessary to elucidate whether the excitatory *NPY* + cells broadly exist across species.

To test whether Exc *NPY* also has distinctive electrophysiological, morphological, and transcriptomic features, we built a standardized pipeline of Patch-seq technique to extract high-quality data from all three modalities from single cells (Supplementary Fig. 9a; see Methods). We performed Patch-seq on inhibitory neurons (*n* = 10 cells), *HPCAL1*+ (*n* = 11 cells), and Exc NPY neurons (*n* = 8 cells) in L2/3 for comparison. We found *SLC17A7*, *HPCAL1*, *CAMK2N1* and *PPP3CA* were enriched in *HPCAL1* L2/3, and *SLC17A7*, *HPCAL1*, *NPY*, *NOV*, *NNAT* and *CRYAB* were enriched in Exc *NPY* (Fig. 4h), consistent with their single-cell transcriptomes (Fig. 4b, f). To further explore the variability in electrophysiological features between *HPCAL1* L2/3 and Exc *NPY*, we compared their electrophysiological features quantitatively. While most features exhibited similarities between cell types, the Exc *NPY* cells have higher input resistance, action potential width, rising time and decay time (Fig. 4i, j; Supplementary Fig. 9b, c and Supplementary Data 3).

### The *OSTN* gene links transcriptomic cell types to a primate-specific neural plasticity mechanism

**Fig. 5:**
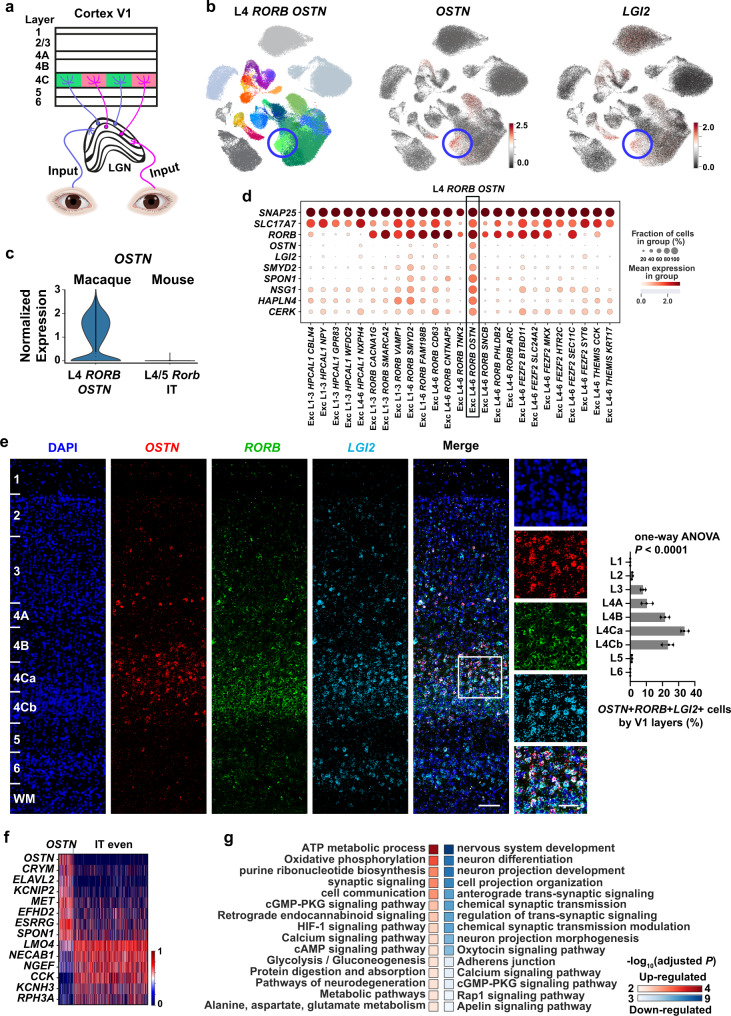
L4 *RORB OSTN* cells are neural activity-induced neurons. **a** Schematic depicting the macaque visual pathway, *OSTN*+ neurons receive sensory inputs from the lateral geniculate nucleus (LGN). Drawings of eyes were created with BioRender.com. **b** Left panel, UMAP plot highlights the L4 *RORB OSTN* cells (Exc L4-6 *RORB OSTN*). Middle and right panel, UAMP plots of the expression of *OSTN* and *LGI2*. Blue circles indicate the L4 *RORB OSTN* cells. **c** Violin plots show the normalized and scaled *OSTN* expression level of macaque L4 *RORB OSTN* and mouse V1 L4/5 IT cortical cells. **d** Dotplot shows the distribution of L4 *RORB OSTN* marker gene-expression across all excitatory cell types. Black rectangle indicates the L4 *RORB OSTN* cells. **e** Left panel, representative RNAscope mFISH images of cells co-expression with *LGI2* (light blue), *RORB* (green) and *OSTN* (red) marker genes from across layers of macaque V1. Scale bars, low magnification, 200 μm; high magnification, 50 μm. Right panel, percentage of positive cells across layers. Data are represented as mean ± s.d. and dots show the data points for individual macaques. The differences between groups were significant, as determined by one-way ANOVA. *n* = 3 macaques. **f** Heatmap of the L4 *RORB OSTN* upregulated and down-regulated genes compared with IT even subclass. **g** KEGG and Biological Process enrichment analysis of the L4 *RORB OSTN* upregulated DEGs (*n* = 200) revealed these genes overlap with oxidative phosphorylation and ATP metabolic process related genes while the L4 *RORB OSTN* down-regulated DEGs (*n* = 200) overlap with cell projection and morphogenesis related genes. *P-*value for each term was determined by Fisher’s exact test implemented in (**g**). Profiler with FDR-adjust for multiple comparisons, indicated as the color-coded squares. Source data are provided as a Source Data file.

The visual experience drives OSTN expression in the primate V1 and affects dendritic arborization and network architecture of neurons^61,77^ (Fig. 5a). Exc L4-6 *RORB OSTN* (*n* = 1364 cells, Fig. 5b), the only cell type in the *IT OSTN* subclass, is a primate-specific cell type that is under activity-dependent regulation, and this cell type features augmented expression of *OSTN* (Fig. 5c). Transcriptomically, we found that L4 *RORB OSTN* is similar to the most abundant and evenly-distributed IT even subclass (Fig. 5b, d), but has its own distinct marker genes (Fig. 5d). One of the marker genes is *LGI2* (encoding leucine-rich, glioma-inactivated protein 2) (Fig. 5b, d). It belongs to the LGI family that participates in the synapse maturation^78,79^. Analysis by RNAscope mFISH reveals that the *OSTN*, *RORB* and *LGI2* mRNA were primarily co-expressed in L4 (Fig. 5e).

Osteocrin (*OSTN*) is a secreted factor that has been repurposed in primates to regulate dendrite growth in activated neurons. The *OSTN* gene expression can be induced by visual experience in primate V1 but not in mice^61,80^, suggesting a primate-specific visual plasticity mechanism. In-depth interrogation of this mechanism in vivo is challenging, due to the lack of counterpart regulatory pathways in rodents. The discovery of the L4 *RORB OSTN* cell types with upregulated *OSTN* expression may contribute to the understanding of primate-specific plasticity mechanisms. For example, the elevated *LGI2* expression in the *OSTN*+ cells may suggest the effect of the *LGI2*-related pathway on suppressing the dendrite growth. The heatmap reveals that the upregulated and down-regulated genes for the IT *OSTN* and IT even subclass (Fig. 5f; Supplementary Data 4). GO and KEGG analyses of *IT OSTN* upregulated genes compared with other IT even subclass (Fig. 5g) highlight their functional involvement in oxidative phosphorylation, ATP metabolic processes and synaptic signaling^77^ (Fig. 5g; left, Supplementary Data 5, 6), suggesting that these neurons are in activated status. The down-regulated genes are associated with neuron projection development and synapse organization, suggesting suppressed morphogenesis in this cell type (Fig. 5g; right, Supplementary Data 7, 8), consistent with the previous finding that OSTN restricts dendrite growth in vitro^61^. Here, functional enrichment analysis revealed an altered cGMP-PKG pathway in the L4 *RORB OSTN* cells (Fig. 5g). Of note, the cGMP pathway is also activated by *Ostn* in the mouse skeleton^81^.

In addition to the excitatory neurons, we found that OSTN expression is also elevated in the Inh L4-6 *PVALB OSTN* (Fig. 3d, e) cell population. This finding extends the OSTN pathway from the excitatory neurons to PVALB neurons and suggests the OSTN induced morphogenesis regulation may be a common mechanism for activity-induced plasticity in neurons. Old World monkeys are an established model for studying bottom-up activity-dependent primary visual cortex plasticity^82,83^. The finding of the L4 *OSTN*-expressing cell type in our dataset suggests the potential of the scRNA-seq technique in understanding the activity-dependent learning and plasticity.

### Conserved and divergent gene-expression patterns between humans and macaques

The transcriptomic homology between humans and macaques (Figs. 2, 3) suggests the similarities in the gene expression across the two species. These results are directly supported by the expression of the canonical marker gene-expression patterns between the two species (Supplementary Fig. 10a). Still, it is worth noting that in order to remove the technical variations across datasets, batch-correction algorithms^84,85^ were applied to the datasets. These algorithms emphasize the shared features across species and may wash out differences between datasets.

To further interrogate the conservation and divergence in gene expression between humans and macaques, we used a supervised classification approach to compare the macaque and human datasets. We trained a classifier using the human cluster information and all shared genes between humans and macaques without batch correction (*n* = 14,516 genes). This classifier was then used to predict the cell annotation of our macaque datasets using a logistic-regression based algorithm^58^. The comparison between our annotation and the predicted annotation was presented in Supplementary Fig. 10b. As expected, both annotation approaches reach very similar results, suggesting that the gene-expression programs are largely shared between the two species.

Transcriptomic differences also exist between species. We compared the expression of neuron differentiation-related genes (*n* = 2006) using Spearman’s correlation-based analysis (see Methods). This comparison revealed genes that have at least twofold changes between humans and macaques (Supplementary Fig. 10c). For the *HPCAL1*+ neurons (*LINC00507*+ neurons in humans), 45 genes were overrepresented in macaques than in humans, including *CLU*, *CAMK2A*, *RAB3A*, and *GRIN1*, while 81 genes were upregulated in humans, including *RBFOX1*, *DLG2*, *LRRTM4*, *NRXN3*, and *PTPRD*. Similarly, the comparisons for the *RORB*+, *FEZF2*+, *THEMIS*+, *PVALB*+, *SST*+, *VIP*+, and *LAMP5*+ neurons also revealed multiple DEGs (Supplementary Fig. 10c). These divergences in the gene-expression profiles suggest the putative transcriptomic differences between humans and macaques.

### Gene set expression patterns are more diverse in excitatory neurons than inhibitory neurons

To systematically assess the extent of gene-expression conservation and divergence across species, and whether different cell types share the same level of transcriptomic divergence, we investigated the expression profiles of selected gene sets by cross-species correlation analysis (see Methods). This analysis was performed between any two species using the same gene set^86^ that exhibits coherent annotations or functions, such as those from a Gene Ontology (GO) term or gene family. This analysis provides a window into the gene-expression profile changes which may not manifest at the whole-transcriptome level^64^. To reduce technical confounders, expression profiles across cells were standardized and subsequently collapsed within the excitatory or inhibitory neuronal category to account for differences in gene-expression scales and cell type representation.

**Fig. 6:**
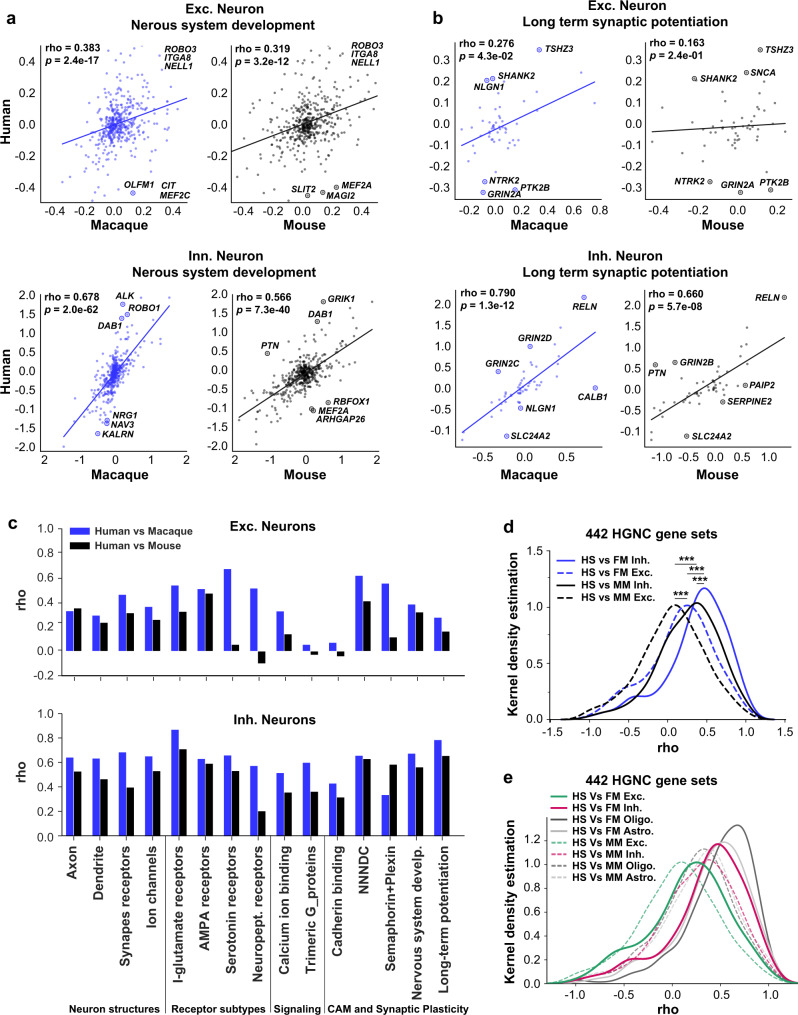
Conserved and divergent gene set expression patterns across species. **a** The nervous system development-related gene-expression level of human excitatory (top row) and inhibitory (bottom row) neurons are plotted against that of macaque (left, blue) and mouse (right, black), using the median z-score of each cell type within species. The top 3 genes with the largest distance to the regression line are labeled in each plot. **b** The long-term synaptic potentiation and its regulation related gene-expression level of human excitatory (top row) and inhibitory (bottom row) neurons are plotted against that of macaque (left panel, blue) and mouse (right penal, black). **c** The correlation coefficient of long-term synaptic potentiation and additional neuron-related gene sets are higher between humans and macaques (blue bars) than between humans and mice (black bars), for 14/15 gene sets tested for excitatory neurons and 14/15 for inhibitory neurons. I-glutamate receptor ionotropic glutamate receptor, Neuropept. receptors Neuropeptide receptors, NNNDC Nrxn/Nlgn/Nphx/Dystroglycan/Cbln, Nervous system develop Nervous system development. **d** The distribution of Spearman’s correlation coefficient (rho) between human (HS) and macaque (FM, blue lines) or mouse (MM, black lines) for both excitatory (Exc., dashlines) and inhibitory neurons (Inh., solid lines), for 442 HGNC gene sets. The correlations between human and macaque are stronger compared with between human and mouse, and are stronger between inhibitory neurons than between excitatory neurons. ***P < 0.001 (8.0e−13, 6.9e−14, 7.0e−5 and 2.1e−4 from the top to bottom) with Kruskal-wallis test followed by Dunn’s test. **e** The distribution of Spearman’s correlation coefficient (rho) between human (HS) and macaque (FM, solid lines) or mouse (MM, dashlines) for excitatory (Exc., green) and inhibitory neurons (Inh., red), and for oligodendrocytes (dark gray lines) and astrocytes (light gray lines) for 442 HGNC gene sets. The correlations for glia (oligodendrocytes and astrocytes) are higher compared with the correlation of neurons. The Spearman’s Correlation’s correlation coefficient (rho) and significance were calculated and labeled within each plot. Source data are provided as a Source Data file.

First, we run cross-correlation using the z-score of the mean expression of the 461 genes in the nervous system development term (GO: 0007399). As expected, the correlation is stronger between monkey and human excitatory neurons than the correlation between mice and humans (Fig. 6a), suggesting that the expression of these genes is conserved within primates but divergent between primates and mice. The same conclusion holds for the inhibitory neurons. Interestingly the correlation coefficient is higher in inhibitory neurons than in excitatory neurons. This is consistent with our observation that the inhibitory neurons are more conservative than excitatory neurons across species. This result is consistent with the previous findings that between humans and mice, the glutamatergic neurons are more divergent than inhibitory neurons in their expression of schizophrenia risk genes^87^.

Next, we examined the expression profiles of the 54 genes from the long-term potentiation gene sets (GO:0060291 and GO:1900271) across humans, macaques and mice. Between human and macaque inhibitory neurons, their correlates significantly (rho = 0.790, *P* < 0.0001) (Fig. 6b), suggesting the gene expression is conserved within primates for this gene set. The correlation between mouse and human inhibitory neurons using the same gene set is also significant (*P* < 0.0001), albeit with a weaker correlation (rho = 0.660) (Fig. 6b). The reduced correlation is consistent with the evolutionary distances among humans, macaques, and mice. This result is also consistent with the previous findings that the expression correlations are highly consistent among primates than in the primate-mouse or primate-ferret comparisons^38^. The same conclusion holds for the excitatory neurons with a stronger correlation between human and macaque (rho = 0.276, *P* = 0.043) than between human and mice (rho = 0.163, *P* = 0.24). Interestingly, focusing on the comparisons between excitatory and inhibitory neurons, we find the gene set expression profile of the same gene set is more similar for inhibitory neurons than that for excitatory neurons (Fig. 6b). This reduced similarity in excitatory neurons suggests the expression patterns for excitatory neurons are more divergent across species than inhibitory neurons in long-term potentiation related genes.

We extended our analyses to additional gene sets, including neuron structures (axon, dendrite, synaptic receptors, and ion channels), neuromodulation (serotonin and neuropeptide receptors), cellular signaling (Calcium ion binding and G-proteins), and cell adhesion molecule (CAM) and plasticity (cell adhesion molecules, nervous system development and long-term potentiation)^64,88^ (Fig. 6c; Supplementary Fig. 11). Out of the 15 gene sets we have compared, 14 exhibited a stronger correlation between human and macaque than that between human and mouse, again recapitulating their evolutionary distance. Notably, the largest differences are not found in the gene sets related to neuron structures, but in the neuromodulation, synaptic connection and plasticity-related ones (Fig. 6c). For example, the correlation for serotonin receptors in excitatory neurons, which play an important role in neuromodulation^89–92^, is not significant between humans and mice (rho = 0.055, *P* = 0.87), contrary to that between humans and macaques (rho = 0.673, *P* = 0.023) (Supplementary Fig. 11a), suggesting a significant shift in gene-expression pattern from rodent to primate. This is consistent with the previous finding that the serotonin receptor was one of the major differences between humans and mice cortical transcriptomes^56^. Another example is the correlation in terms of the cadherin binding gene set (GO: 0045296, *n* = 69 genes). Cadherins (calcium-dependent adhesion) are a type of CAMs important for synapse formation and plasticity^93,94^. Their cross-species divergence is much higher in excitatory neurons than that in inhibitory neurons (Fig. 6c). The same observations hold true for the Neurexins/Neuroligins/NphX/Dystroglycan/Cbln^38,64,95–102^ genes (*n* = 19), and semaphorin and plexin-related gene sets (*n* = 22)^64,88^, all of which play crucial roles in synapse plasticity and formations.

We ran the same analysis for each of the gene sets from the 3888 GO terms and 442 HGNC (Human Genome Nomenclature Committee) gene families^64^, and demonstrated the global distribution of the correlation coefficients of these terms among the three species for both excitatory and inhibitory neurons (Fig. 6d, e; Supplementary Fig. 11e, f). As expected, the correlations between humans and macaques (blue lines) are significantly higher than those between humans and mice (black lines) (Fig. 6d; Supplementary Fig. 11e). Moreover, adding to our earlier observation based on selected gene sets, the correlations between excitatory neurons are lower than that of inhibitory neurons (*P* < 0.0001), suggesting the excitatory neurons are more divergent than inhibitory neurons. Compared with excitatory neurons, analysis of astrocytes or oligodendrocytes yields higher interspecies correlation for gene sets from the nervous system development-related pathway (Supplementary Fig. 11d), the 3888 GO term (Supplementary Fig. 11e, f) and 442 HGNC (Fig. 6d, e) gene families. These results provide further evidence that excitatory neurons could be a major source of divergence between primates and rodents.

## Discussion

In this study, we established an in-depth single-cell atlas for the primary visual cortex, which is the most studied non-human primate brain area. Although several datasets for primates have been generated for both developing^14,15^ and mature primates^14,15^ using single-cell or single-nucleus RNA-sequencing approaches, our single-cell transcriptomic atlas constructed from whole cells of the V1 provides additional insights to the field. The large number of cells dedicated to one brain area, together with the transcriptomes recovered from whole cells, provide a valuable resource. In addition to the cell types that have been established^14,15,31,38,56^, our dataset, with deeper sequencing of more cells, captures rare cell types for further interrogations of the primate neocortex. Detailed annotation of our dataset for both excitatory and inhibitory cell types, combined with the homology analysis across datasets, revealed novel cell types, such as the excatory NPY cells (Exc *NPY*) and primate-specific activity-depended IT cells (IT *OSTN*), potentially important in sensory modulation and plasticity. Also, our macaque atlas provides an intermediate between humans and mice, further demonstrating that the excitatory neurons are evolutionarily more diverse than inhibitory neurons or glial cells across species.

It is worth noting that in order to preserve as many cell types as possible, we chose to select cells using a relatively low number of detected genes threshold (keeping cells with more than 200 detected genes) around the studies alike^38,39,103,104^. The selection of the threshold had very limited effects on the dataset where the cells with fewer than 500 detected genes only comprise 1.9% of the total population in our dataset. The cells with fewer than 800 detected genes only comprise 6.9% of the total population (Supplementary Fig. 1j). Several cell types do contain a fraction of neurons that have a lower number of uniquely detected genes (Supplementary Fig. 1k).

Isolation of high-quality live neurons from the adult cortex is one of the major bottlenecks for the broad application of scRNA-seq^9,105^. It is particularly challenging for adult primates. To achieve a high survival rate for neurons, we incorporated knowledge accumulated from multiple fields to ensure good isolation of neurons while maintaining their viability^106–108^. The animals are perfused with a cold anti-coagulating perfusion solution to remove hematocytes. A cocktail of neural activity inhibitors is added during perfusion and dissociation to suppress neural activity^36^. We also utilized adult brain slicing protocols for in vitro patch-clamping^109,110^, in which brains are sliced within a neural protective recovery solution to enhance neuronal survival. This approach helps to disentangle the neurons and reduces the intensity of cell-digestion. With these modifications, we successfully established a dataset with a limited amount of hematocytes and a neuron ratio that is comparable with that seen in vivo.

This dataset could be useful for elucidating the developmental origin of certain cell types. The LAMP5-LHX6 cell type, for example, is also found in humans, marmosets and mice^31,38,56^. The macaque Inh L1-6 *LAMP5 LHX6* cells are integrated well with human and mouse *LAMP5 LHX6* cells that were also transcriptomically similar to the hippocampal neurogliaform cells^38,111^. Emerging evidence suggests that, besides MGE and CGE, there can be a third source of cortical inhibitory neurons^38,112^. In mice, *LHX6* and *ADARB2*, which marks the MGE- and CGE-originated inhibitory neurons respectively, are co-enriched in the Preoptic Area-derived (POA) neurogliaform cells^112^. Macaque Inh L1-6 *LAMP5 LHX6* cells may correspond to this cell type^38^.

Taking advantage of this dataset, we also identify and further experimentally validate two plasticity-related cell types that have not been reported previously. One is an excitatory *NPY*+ cell type (Exc *NPY*) that is closely related to L2/3 IT cells, but expresses the neuromodulator *NPY*. This cell type also expresses *DRD3*, which encodes a D2-like dopamine receptor that mediates learning processes^113^. It regulates the remodeling of brain structure and is a classical candidate gene for susceptibility to schizophrenia^114^. In particular, mutation of the *DRD3* gene disturbs eye movement in schizophrenic patients^115^. Our findings of the *DRD3*-expressing excitatory *NPY*+ cells may serve as a bridge in understanding the experience-dependent plasticity of V1, and suggest a tentative mechanism for top-down regulation of adult learning. To the best of our knowledge, this is the first study characterizing an excitatory *NPY* cell type. A combination of scRNA-seq, patch-clamp, and RNAscope mFISH technique suggests that it is a distinct excitatory cell type located in the L2/3 of the primary visual cortex. This cell type shares many similar electrophysiological properties with L2/3 excitatory neurons. Due to the technical difficulties and rarity of this cell type, a complete interrogation of this cell type is far from complete. For example, a thorough investigation of its morphology is needed, which is currently hard to achieve due to the rarity of this cell type and the absence of proper transgenic lines in macaques. Also, it is still unknown whether this cell type is macaque-specific or broadly exists across species. Immunostaining studies combined with patch-seq experiments using humans and mice are necessary to examine its existence across species. A complete understanding of the Exc *NPY* and its role in visual signal processing may contribute to our understanding of visual cognition.

We also identified a primate-specific excitatory cell type, expressing the markers *RORB* and *OSTN*. This cell type matches the description of the reported activity-induced L4 *RORB*+ cells found specifically in anthropoid primates^61^. The morphogenesis of these cells is restricted, but the mechanism underlying this is unknown. Here, detailed transcriptome information reveals that the restrictions could be mediated through the cGMP and Rap1 pathway. This finding suggests a tentative primate-specific bottom-up mechanism for visual plasticity. Currently, this mechanism is hard to validate in primates. The development of transgenic tools in primates is crucial and may enhance our understanding of these mechanisms in future.

While primates can handle more complicated tasks with higher flexibility^116^, mammals from mice to primates utilize similar building blocks for sensory information processing^117^. Although core conserved molecular identities exist for cortical neuron cell types across species^39,86^, single-cell RNA sequencing evidence suggests gene expression and the abundance in each cell type underwent substantial evolutionary changes for inhibitory neurons^38^. Compared with the inhibitory neurons, evidence suggests a more diverse program for excitatory neurons between reptiles and humans^86^. Here, the comparisons using mice, macaques and humans extend the findings and provide evidence that glutamatergic neurons are more diverse than inhibitory neurons, astrocytes and oligodendrocytes. Further, our comparisons suggest that instead of novel cell types or altered cell-intrinsic properties such as receptor and ion channel types, it is the enhanced modulatory system, together with the activity-dependent synaptic plasticity in excitatory neurons that differs between primates and rodents. This activity- and experience-dependent network plasticity may play a critical role in enabling primates to develop complicated cognitive abilities.

## Methods

### Ethical compliance

All experimental procedures were approved by the Institutional Animal Care and Use Committee (IACUC) at Zhongshan Ophthalmic Center, Sun Yat-sen University, China. This study was consistent with the Principles for the Ethical Treatment of Non-Human Primates.

### Macaques

All macaque monkeys (*Macaca fascicularis*) were obtained from the Blooming-Spring Biotechnology Co. Ltd (Guangdong, China) or generously provided by the other laboratories in Zhongshan Ophthalmic Center, Sun Yat-sen University, China. Macaques were raised in standardized conditions with a 12 h (h) light, 12 h dark schedule, and given regular diets three times a day and careful veterinary oversight. Macaques were housed for 2 weeks before experiments in the institutional animal facility. All macaque monkeys tested negative for tuberculosis, simian immunodeficiency virus, herpes B virus, simian T-cell lymphotropic virus, simian type D retroviruses, etc. Before the experiment, no brain disease was reported in these macaques.

### Tissue procurement

The primary visual cortex (V1) was sampled from fresh brains of eight macaques (4–15 years old, one male and seven females) in the study. Briefly, the macaques were deeply sedated with isoflurane and then euthanized with an overdose of pentobarbital. The macaques were transcardially perfused with 2 liters of cold carbogen-bubbled artificial cerebrospinal fluid (ACSF) (as described below) to reduce the internal temperature of the entire brain from “inside-out”. The brains were carefully moved from the skulls, and V1 tissue was isolated and submerged in fresh ice-cold carbogen-bubbled NMDG solution (as described below). The position of the V1 was referred to as the “A Combined MRI and Histology Atlas of the Rhesus Monkey Brain”^118^. V1 blocks from each macaque were sectioned into 300 μm thick slices and processed for single whole-cell suspension preparation as described below. To characterize histological hallmarks of V1, the adjacent sections were sectioned into 50 μm thick, and fixed in 4% paraformaldehyde (PFA) for Nissl staining. The V1 regions of interest were fixed in 4% PFA and processed for fluorescent in situ hybridization (FISH) and immunofluorescence.

### Tissue processing

The experimental procedure was optimized for preserving transcriptional state. In order to improve adult macaque neuronal survival, we updated our artificial cerebrospinal fluid (ACSF) and N-methyl-d-glucamine (NMDG) solution formulation following previously reported methods^108,109^. Our NMDG solution consisted of 93 mM NMDG (Sigma, M2004), 30 mM NaHCO_3_ (Sigma, S5761), 1.2 mM NaH_2_PO_4_ (Sigma, S5011), 2.5 mM KCl (Sigma, P9541), 20 mM HEPES (Sigma, S5011), 25 mM D-Glucose (Sigma, G8270), 5 mM Sodium Ascorbate (Sigma, A4034), 2 mM Thiourea (Sigma, T8656), 3 mM Sodium Pyruvate (Sigma, P8574), 10 mM MgSO_4_ (Sigma, M7506), 0.5 mM CaCl_2_ (Sigma, C4901), 93 mM HCl (Guangzhou chemical reagent factory, CB11-TD). The ACSF used to tissue preparation and cell isolation procedure consisted of 125 mM NaCl (Sigma, RES0926S-A7), 25 mM NaHCO_3_ (Sigma, S5761), 1 mM NaH_2_PO_4_ (Sigma, S5011), 2 mM KCl (Sigma, P9541), 25 mM D-Glucose (Sigma, G8270), 2 mM CaCl_2_ (Sigma, C4901), 1 mM MgCl_2_ (Sigma, M1028), 5 mM Sodium Ascorbate (Sigma, A4034), 3 mM Sodium Pyruvate (Sigma, P8574), 0.01 mM Taurine (Sigma, T8691), 2 mM Thiourea (Sigma, T8656), 1 mM Kynurenic Acid (Sigma, K3375), 10 μM CNQX (Tocris, 1045), 100 μM DL-AP5 (Tocris, 0105), and 1 μM Tetrodotoxin (Tocris, 1078), bubbled with a carbogen gas (95% O_2_ and 5% CO_2_) and with a PH of 7.3–7.4. The V1 blocks were sectioned in ice-cold NMDG on a vibratome (Leica VT VT1200S) into 300-μm slices. The slices of interest were immediately transferred to a cell culture dish containing cold carbogen-bubbled ACSF. The layer-enriched or multiple layers of V1 were microdissected under a dissection microscope with a cooled platform, and dissected tissue pieces transferred to other cold carbogen-bubbled ACSF for dissociation. To collect layer-enriched samples, we seperated the slices based on the location of layer 4, which were clearly visible under the dissection microscope (Leica S9E), using sterile 30 gauge needles (KINDLY Group, KDL0010).

### Single whole-cell suspension preparation

We generated single-cell suspensions using an in-house procedure. The dissociation solution included 30 U/mL papain (Worthington, LS003127), 0.05% trypsin-EDTA (Gibco, 25300054) and 2.5 mg/mL DNase I (Sigma, D5025) in ACSF warmed at 34 °C for 10 min before use. The tissue pieces were placed into the dissociation solution and incubated for about 1 h at 34 °C continuously bubbled with carbogen. After incubation, the dissociation solution was pipetted out of the tube and the cold carbogen-bubbled ACSF containing 10% fetal serum albumin (Gibco, 10099141C) was used to stop digestion. The tissue pieces were dissociated into single cells by gentle trituration BSA-coated Pasteur pipettes, which were fire-polished with successively smaller bores. Following trituration, the cells were filtered through a 40 μm filter (BD Falcon, 352350) to eliminate cell clumps. The dead cells and debris were removed by using Dead Cell Removal Kit (MACS, 130090101), and the cells were washed twice and resuspended in PBS (Gibco, C1001050BT) with 0.01% BSA (Sigma, B2064). The cells were processed for analyzing cell number and viability using an automated cell counter (Bio-Rad, TC20) and further diluted to desired concentrations for scRNA-seq. During all steps the cells were kept on ice or at 4 °C except for enzymatic digestion. Cell suspensions with a cell viability of >90% were used for scRNA-seq library and sequencing.

### Single-cell RNA-sequencing (10× Genomics)

scRNA-seq experiments were conducted on the Chromium Single-Cell platform (10× Genomics). Cells barcoding, cDNA synthesis and libraries construction were using single-cell gene-expression 3′ kit Version 2 (10× Genomics, PN120237) or single-cell gene-expression 3′ kit Version 3 (10× Genomics, PN1000075) according to the manufacturer’s instructions. Briefly, the cell suspension was mixed with master mix, gel beads and oil were loaded on the Chip wells to generate single-cell Gel Bead-In-Emulsions (GEMs). The captured cells released RNA, reverse transcribed and synthesized full-length cDNA, which amplified to enough the cDNA for library construction. The quality of cDNA was checked using the High Sensitivity DNA Kit (Agilent Technologies, 5067-4626) on Agilent Bioanalyzer 2100 (Agilent Technologies, G2939BA). The good-quality cDNA underwent enzymatic fragmentation, end repair and size selection to optimize the cDNA size. Then adaptor ligation, sample index PCR and sided size selection was performed to generate Single-cell libraries. Libraries were sequenced on the Illumina Novaseq 6000 platform.

### Nissl staining

Tissue was coded to prevent experimenter bias, stained with NeuroTrace^TM^ 530/615 Red Fluorescent Nissl Stain kit (Invitrogen, N21482) and Cresyl Violet acetate (Sigma, C5042-10G), respectively. Sections were fixed 4% PFA at 4 °C for 1 h, and hydrated through a graded series containing 10%, 15% and 30% sucrose, then rinsed in PBS for three times. For Fluorescent Nissl Stain method, the sections were permeabilized in 0.1% TritonX-100 for 10 min, washed with PBS for 5 min. Then the sections were incubated with Neuro Trace stains for 20 min, washed with PBS for 10 min, and mounted with DAPI (Abcam, ab104139). Images were acquired with a laser scanning confocal fluorescence microscope (Zeiss LSM880). For Cresyl Violet acetate staining, the sections were stained with 1 mg/mL cresyl violet solution for 10 min, dehydrated twice in 70% alcohol 5 min, dehydrated twice in 95% alcohol for 2 min, cleared twice in xylene 5 min, and mounted with permanent mounting medium. Sections were imaged using an advanced research microscope (NIKON ECLIPSE 80i).

### Golgi staining

For the neuronal morphology visualization slides, slices were stained with Golgi-Cox staining. Impregnation solution preparation is prepared as follows: Solution A including 5% solution of Potassium dichromate (K_2_Cr_2_O_7_, Guangzhou chemical reagent factory, AD22), Solution B including 5% solution of Mercuric chloride (HgCl_2_, Guangzhou chemical reagent factory, YA42), and Solution C including 5% solution of Potassium chromate (K_2_CrO_4_, Guangzhou chemical reagent factory, AC10). Mix 5 mL solution A, 5 mL solution B, 4 mL solution C and 10 mL distilled water to get Golgi-Cox solution. After sufficiently mixing the solution, it was kept in the dark at room temperature for 3 days. After reddish precipitates form, aspirate the supernatant to get the working solution. Slices were treated with a working solution, and protected from exposure of light whenever possible. After 10 days of impregnation, transfer slides into the 30% sucrose (BioFroxx, 1245) solution for 6 h. The slides were incubated in 14% ammonia solution (Aladdin, A112077) for 10 min in the dark by gently shaking them. We dehydrated the slides through 70%, 80%, 95%, and 100% (twice) ethanol for 5 min each, transferred the slides to the xylol (Macklin, X820585) for 10 min, then mounted the sections with a permanent mounting medium. Images were obtained with an advanced research microscope (NIKON ECLIPSE 80i).

### Immunofluorescence

The fresh brain tissue was fixed for overnight in 4% PFA in PBS at 4 °C, rinsed in PBS and dehydrated in 30% sucrose in PBS at 4 °C. Dehydrated tissue embedded and frozen at −80 °C in optimum cutting temperature compound (OCT; Tissue-Tek) and sectioned at 30μm using cryostat microtome (Leica CM1950). Slices were washed with PBS, blocked with 5% BSA, 1% Normal Goat Serum (Jackson ImmunoResearch, 005000121) and 0.3% Triton X-100 PBS for 1 h at room temperature, followed by incubation with the primary antibodies overnight at 4 °C. The primary antibodies used were anti-SST (rat, Millipore, MAB354, 1:50), anti-NOS1 (rabbit, CST, 4231S, 1:200), NR2F2 (mouse, Abcam, ab41859, 1:200). Sections were washed three times in PBS and incubated with secondary antibodies at room temperature for 1 h. The secondary antibodies used were Alexa Fluor 488 anti-Rat (Invitrogen, A11006, 1:1000), Alexa Fluor 594 anti-Rabbit (Invitrogen, A11037, 1:1000), and Alexa Fluor 594 anti-Mouse (Invitrogen, A-11032, 1:1000). Sections were washed three times in PBS and stained with DAPI (Abcam, ab104139). Slides were imaged with a confocal microscope (Zeiss LSM 880) and analyzed using Zeiss ZEN software suites (blue edition). Images were acquired with 1 AU pinhole size using 20x or 63x objectives at the resolution of 1024×1024 pixels. Stacks of optical slices were collected through the entire z-axis of each slice.

### RNAscope mFISH

To verify the interesting genes from scRNA-seq datasets, we collected small tissue blocks, adjacent to the tissue sample dissected, for RNAscope multiplex fluorescence in situ hybridization (mFISH). RNA FISH was performed using RNAscope Multiplex Fluorescent Reagent v2 kits (Advanced Cell Diagnostics, 323100) according to the manufacturer’s recommended protocols. The fresh tissue blocks were then fixed in 4% PFA for overnight, dehydrated in 30% sucrose in PBS, embedded in OCT and sectioned for 20-μm brain sections, collected on SuperFrost Plus glass slides (ThermoFisher Scientific). Proprietary probes (Advanced Cell Diagnostics) were as follows: *SLC17A7* (415611), *HPCAL1* (846051-C3), *NXPH4* (1000961-C2), *DRD3* (529681), *NPY* (416671-C2), *GAD1* (404031-C2), *LAMP5* (487691-C3), *NKX2-1* (468991), *LHX6* (460051), *PVALB* (422181-C3), *OSTN* (876281), *RORB* (876303-C3), *SST* (310591), *UNC5B* (475161), *LGI2* (1109831). Positive controls Probe-NHP POLR2A, PPIB and UBC (Advanced Cell Diagnostics, 320901) and negative control Probe DapB (Advanced Cell Diagnostics, 320871) were used on each tissue sample to assess RNA quality. Following hybridization and amplification, Nuclei were labeled by DAPI and coverslips were mounted with ProLong Gold Antifade Mountant (ThermoFisher Scientific, P36930). Images were acquired at 20x, 63x, 100x objectives on a confocal microscope (Zeiss LSM 880). Images were processed and analyzed using Zeiss ZEN software suites (version 23) and Adobe Photoshop software (version 21). Counts were conducted on sections from more than 3 macaque tissues.

### Single-cell RNA sequencing data processing

Cell Ranger (10x Genomics, version 3.0.2) was used to process Chromium single-cell 3’ RNA-seq output. Briefly, the raw base call files were demultiplexed into FASTQ files using the “cellranger mkfastq” command. The ensembl Macaca fascicularis 5.0 genome and ensembl genome annotation file (GTF) was downloaded from Ensembl^119^ to build a custom Macaca fascicularis reference, using the “cellranger mkref” command. Reads were demultiplexed to fastq files based on the index with the “cellranger mkfastq” command. The FASTQ files were aligned to the ensembl Macaca fascicularis reference to generate a data matrix using the “cellranger count” command.

For quality control, cells with fewer than 200 features and cells with higher than 30% mitochondrial contents were excluded from the analysis. Features expressed in fewer than 10 cells were also excluded. Data analysis and visualization were conducted using SCANPY (version 1.4.6)^120^. We excluded the doublets in the datasets using Scrublet (version 0.2.1)^121^. The doublets were determined on a cluster basis. The clusters were labeled as doublets and removed from further analysis if: 1) the cluster showed obvious elevation of the scrublet score in the dimension reduction visualization (such as t-SNE or UMPA) of the dataset (see below for the demotion reduction method); and 2) the cluster shares the marker gene-expression pattern of two or more clusters.

We mainly used SCANPY^120^ for data analysis and visualization with following steps performed in order: data normalization, log-transformation, highly variable genes (HVG) selection and principal component analysis (PCA). Expression levels were normalized to counts per 10,000 (NC). That is, the total mapped exonic reads of a gene within one cell is scaled by the total number of all mapped reads of that cell and times 10,000. This number will be 1/100 of counts per million (CPM). The normalized counts were then transformed by log_2_ (NC + 1) and used for further analysis, unless indicated otherwise. Highly variable genes were selected based on the log-transformed data. The principal component analysis was run with the selected high variable genes. If needed, the HVG and PCA were re-calculated for subsets of cells.

To integrate data from different samples and 10x experiment versions, Harmony (version 0.0.5)^84^ was used on the PCA embedding with each sample. This correction was sufficient to accommodate multiple technical and biological factors, such as platform, batch, age and sex.

Based on the Harmony-corrected latent space, T-distributed stochastic neighbor embedding (t-SNE)^122–124^ or Uniform Manifold Approximation and Projection (UMAP)^125^ and Louvain^126^ clustering were performed using SCANPY. Firstly, the neighborhoods were calculated with scanpy.pp.neighbors, using 15 local neighborhoods and all 50 of the Harmony-corrected PCAs for the knn graph. The connectivities were computed using the ‘umap’ or ‘tsne’ method with Euclidean distance. Then UMAP embedding was calculated using ‘scanpy.tl.umap’ with the minimal effective distance of 0.5, the spread of 1.0, the initial learning rate of 1.0, negative sample weighting of 1.0, and negative edge sample rate of 5. The Louvain clustering was calculated using sc.tl.louvain on the neighborhood calculated in the previous steps, with the resolution of 2, with the vtraag package, and no weights from knn graph. Several clusters were further divided using the resolution ranging from 1 to 5. SCCAF (version 0.0.10)^58^ was used to determine the self-consistency of the clusters using the SCCAF_assessment function. 200 cells per cluster were picked for 5-fold cross-validation training of the classifier and the remaining cells were used to test for segregation accuracy. Clustering with an accuracy on the hold-out set > 84% was accepted. SCANPY, as well as seaborn, were used for data visualization of heatmap, violin plot, dendrogram, and diffusion maps.

Differentially-expressed genes (DEGs) for each population were selected by MAST, which used the hurdle model^127^ to remove the technical effects. The top 200 DEGs for the cell population of interest were selected for Gene Ontology (GO)^128,129^ and Kyoto Encyclopedia of Genes and Genomes (KEGG) enrichment analysis^130–132^ using gProfiler (version 1.0.0)^133^ or Gene Set Enrichment Analysis (GSEA)^134,135^.

The data projection from human data to macaque data was performed with SCCAF^58^ where logistic-regression models were trained on all the shared genes between the two species and projected to the other species.

### Human space transcriptome data analysis

Adult human cerebral cortex space transcriptome dataset (V1_Human_Brain_Section_1—Adult Human Brain 1, Cerebral Cortex, Unknown orientation) was downloaded from the 10× Genomics. The dataset was analysed and visualized following the standard pipeline provided by SCANPY. Briefly, wells with the counts range from 2,000-35,000, and mitochondrial contents lower than 20% were kept for further analysis. Features expressed in fewer than 10 wells were excluded. Data were normalized, log transformed and HVGs were selected for the calculation of PCA. UMAP and Louvain clustering were performed. And the expression of selected genes was visualized.

### Cross-species cell-type homology

We aligned the macaque V1 data with the human cortex^56^ data and mice cortex^31^. We downloaded the orthology genes between human and macaque and between mouse and macaque from Ensembl. Only the uniquely mapped orthology genes were kept in the comparison. Then we estimated the cell-type homology following a published method^56^ with slight modification. Briefly, we separated cells into excitatory neurons, inhibitory neurons and non-neuronal categories. For each category and species, we picked 500 cells (or all of the cells with populations with cell numbers < 500) for each cluster at the subclass level with necessary adjustment to coordinate the cell types between the two species. To integrate the macaque dataset with human dataset for species comparison, we picked up to 2000 HVGs and aligned the dataset using the Harmony algorithm. At the Harmony-corrected PCA space, we calculated the nearest neighbors based on the Euclidean distance of each sample. Then we clustered the Louvain community detection on the Euclidean distance. The overlap was defined as the sum of the minimum proportion of samples in each cluster that overlapped within each Harmony cluster. Cluster overlaps were visualized as heat maps.

We used a similar pipeline to integrate the macaque dataset with mouse dataset, except for adding integrated the datasets using the single-cell variational inference (scVI) method^85^ before Harmony^84^ to account for the batch effect. The scVI uses continuous covariates including n_genes and n_counts and categorical covariate of species for the correction. The model was trained for 600 times for fully converged training results. The scVI latent space was used to calculate the Harmony-corrected PCA. The UMAP and louvain clustering was then calculated in the Harmony-corrected PCA space.

### Reference datasets

#### Hodge dataset

This is a human cortical dataset from multiple brain areas^56^. We downloaded the raw expression matrix and the cell type annotation from (https://portal.brain-map.org/atlases-and-data/rnaseq). The annotations in the downloaded dataset were used as the cluster names for the cross-species alignment analysis. The dataset was analyzed and visualized with default SCANPY analysis workflow.

#### Han dataset

This is an adult macaque (*Macaca fascicularis*) dataset from multiple organs^16^. We downloaded the raw expression matrix together with the cell type annotation from (https://db.cngb.org/nhpca/download). The dataset was analyzed and visualized with default SCANPY analysis workflow.

#### Schmitz dataset

This is a developing macaque (*Macaca mulatta*) dataset of inhibitory neurons^15^. We downloaded the raw expression matrix together with the cell type annotation from (https://www.ncbi.nlm.nih.gov/geo/query/acc.cgi?acc=GSE169122). The dataset was analyzed and visualized with default SCANPY analysis workflow.

#### Tasic dataset

This is a mouse cortex dataset^31^ with pyramidal neuron projection information. We downloaded the raw expression matrix together with the cell type annotation from (https://portal.brain-map.org/atlases-and-data/rnaseq). The dataset was analyzed and visualized with the default SCANPY analysis workflow.

#### Zhu dataset

This is an adult macaque (*Macaca mulatta*) dataset^14^. We downloaded the dataset from (http://www.evolution.psychencode.org/). The dataset was analyzed and visualized with default SCANPY analysis workflow.

### Cross-species gene-expression comparison

To quantitatively assess the similarity of the gene-expression architecture across species, we performed the cross-correlation of human gene expression with that of macaques or mice using genes of selected genesets. We used the z-score of the gene-expression level instead of counts to minimize the batch effect. The z-scores for each gene in each cell are calculated by normalizing, log-transforming and scaling of the gene counts, following the standard SCANPY analysis pipeline^120^. To reduce the sampling bias, we firstly calculated the mean z-score within each cell subclass and then calculated the mean of the mean z-score of all subclasses for excitatory or inhibitory neurons respectively. For each comparison, we used all the genes from the GO terms of interest. The Spearman’s rank correlation coefficient (rho)^136,137^ and P value was calculated using the scipy.stats.spearmanr package. To visualize the complete distribution of the correlations, we in addition calculated the 3888 GO gene sets and 442 HGNC gene sets established for mice transcriptional architecture study^138^ and converted them to human or macaque genes using the orthology genes downloaded from Ensembl. The distribution differences among categories are calculated using the nonparametric Kruskal-Wallis test followed by Dunn’s test for the pairwise comparisons using the Python scipy.stats.posthoc_dunn function for Bonferroni adjustments for *P* value.

### Preparation of brain slices for Patch-seq

The brain slices were obtained from 4–6 years old macaque monkeys (*Macaca fascicularis*). macaques were sedated with ketamine (10 mg/kg), and then intubated and maintained throughout the whole surgery with isoflurane and oxygen. The brain was then obtained and primary visual cortex slices (300 μm) were prepared using a vibratome. Slices were incubated in the bubbled NMDG solution at 34 °C for 10 min and then transferred to the oxygenated ACSF at 34 °C for 1 h. The slices were kept to recover in oxygenated ACSF at room temperature (22 °C) until recording. In order to maintain the neuron physiological and morphological characteristics, the ACSF was used to patch with some modifications without 5 mM sodium l-ascorbate, 3 mM sodium pyruvate, 0.01 mM taurine, 2 mM thiourea, 1 mM kynurenic acid (Sigma, K3375), 100 μM DL-AP5 (Tocris, 0105), and 1 μM tetrodotoxin (Tocris, 4368-28-9). Importantly, NMDG solution and ACSF equilibrated in 95% O2 and 5% CO2 was used in all steps.

### Whole-cell recording and single-cell sample collection

To simultaneously obtain electrophysiology and transcriptome data from the same neurons, we applied our developed Patch-seq protocol142,143 with minor modifications. All work surfaces and equipment were clean with ddH2O, 75% ethanol, DNA-OFF and RNase Zap, and all solutions in contact with the RNA sample were prepared under strict RNase-free conditions. The recording chamber was perfused with ACSF and exchanged for 5 min before mounted slices. The tissue slices were placed in the recording chamber and the target cells were visualized under differential interference contrast microscope (Olympus) at 40x magnification. The recording pipettes were filled with 0.3 μL RNase-free an intracellular solution consisting of 111 mM gluconic acid, 4 mM KCl (Sigma, P9541), 10 mM HEPES (Sigma, H3375), 0.2 mM EGTA (Sigma, E3889), 4 mM Mg-ATP (Sigma, A9187), 0.3 mM Na-GTP (Sigma, G8877), 5 mM Na-phosphocreatine (Sigma, P7936), 13 mM biocytin (Sigma, B4261), 1U/μL recombinant RNase inhibitor (Takara, 2313 A), pH7.3. Cells were patched with RNase-free recording pipettes (5-8 MΩ resistance) in whole-cell mode. After the seal resistance value was more than 1 GΩ, the cells were held at −70 mV and break-in with negative pressure. Recording was collected using MultiClamp 700B amplifier (version 11.2) and HEKA Patchmaster software (version 2.65) with the desired electrophysiology protocol. The whole-cell recording was held for 20-30 min in order to allow biocytin diffusion into the complex dendritic arborization and distal axonal branches. After recordings, the cell content including cell nucleus was visibly extracted from the cell body using negative pressure. The pipette was carefully removed and content of the harvest pipette was immediately injected into a 0.2 mL PCR tube containing 4 μL freshly-prepared lysis buffer. Our lysis buffer consisted of 1 U/μl of recombinant RNase inhibitor (Takara, 2313 A), 0.33%Triton X-100 (Sigma, T8787), 0.5 mM dNTP (Thermo Scientific, R1121), 2.5 μM oligo-dT primer.

### cDNA amplification and library preparation for Patch-seq

We performed single-cell RNA sequencing following the Smart-seq2 protocol^139^. The purified full-length cDNA were quantified using the Qubit High Sensitivity DNA assay (ThermoFisher Scientific, USA) and the size distribution of the cDNA was determined using the High Sensitivity DNA Kit (Agilent Bioanalyzer, 5067-4626) on Agilent Bioanalyzer 2100. Samples containing less than 0.2 ng/μL cDNA (15 μL volume) and with an average size less than 1500 bp were not constructed library. The cDNA libraries were prepared using Nextera XT DNA Library Preparation (Illumina, FC-131-1096) and NexteraXT Index Kit V2 Sets A (Illumina, FC-131-2001) according to manufacturer’s instructions. The pooled cDNA libraries were checked using an Agilent Bioanalyzer 2100. Libraries were sequenced on an Illumina Novaseq 6000 instrument with a sequencing setup of 150 -base-pair paired-end reads.

### Extraction of electrophysiological features

We developed a custom-made MATLAB software kit to analyze the electrophysiology data offline using MATLAB (version 9.7). The electrophysiological properties of the neurons were automatically extracted using a similar method as previous studies^140,141^. To extract the properties of action potential (AP), we used the first AP fired by our step stimulation protocol. To calculate the threshold of the AP, the trace within 20 ms preceding the AP peak was used to calculate the third derivative first and the data point with the maximal third derivative value was defined as the threshold of the AP. The AP amplitude was the difference between the threshold potential and the peak potential of the AP. The AP width was calculated as the AP duration at the membrane voltage halfway between AP threshold and AP peak. The time range from the threshold point to the action potential peak is ‘AP rising time’ while ‘AP decay time’ is the time range from the action potential peak to the potential equal to the threshold. The afterhyperpolarization (AHP) was defined as the voltage difference between the threshold and the minimal value of the trough after the AP. The rheobase refers to the minimum stimulation current to elicit any action potentials. The time range from the stimulation onset to the threshold of the first AP at the rheobase sweep was defined as the spike delay time. We calculated the sag ratio and rebound voltage using the hyperpolarizing trace elicited by the maximal negative current injection. The sag ratio was defined as the difference between the sag trough of the trace and the resting membrane potential (RMP), divided by the difference between steady-state membrane voltage and the RMP. The trace from the stimulation offset to the peak of the depolarizing trace following the offset was fitted with an exponential function, and the potential difference between the maximal value of the fitting curve and the RMP was defined as the rebound value. The time constant τ (tau) was computed as the time constant of the exponential fit to the hyperpolarizing trace (from the stimulation onset to the minimal potential within the first-half stimulation period). To calculate the adaptive index, the inter-spike intervals were first measured, and then the ratio of the last and the first inter-spike intervals was calculated. For each hyperpolarizing current injection, input resistance was calculated as the ratio of the steady-state membrane potentials deflection to the corresponding injected current value (we took the average membrane potential of the last 100 ms before stimulus offset as steady state). The median of these values over all hyperpolarizing traces was taken as input resistance.

### Single-cell RNA-sequencing data processing for Patch-seq

Raw sequencing reads were checked using FastQC for sequence quality, base quality, GC content, base content, sequence duplication levels, overrepresented sequences, contamination and so on. Reads were trimmed with Trimmomatic to remove the contaminant adapters and low-quality bases or reads to achieve clean data. The reference genome was prepared using the ensembl Macaca fascicularis 5.0 genome and ensemble genome annotation file. Reads were aligned to the reference genome (assembly) using STAR (version 2.7.10a) with default settings. Quantification was performed using the RSEM program.

### Histology and morphological reconstruction

Following electrophysiological recordings and single-cell RNA harvest, the slices were immediately fixed in freshly-prepared 2.5% glutaraldehyde, 4% paraformaldehyde solution in 0.1 M phosphate buffer (PB; pH = 7.4) at 4 °C in order to improve the staining quality. After 14 days of fixation, the slices were subsequently processed with the avidin-biotin-peroxidase protocol as reported studies^142,143^. Briefly, slices were several washes with 0.1 M PB, digests endogenous peroxidase in 3% H_2_O_2_ for 30 min, several washes with 0.1 MPB and incubated 5% Triton X-100 with ABC Elite kit (Vector Laboratories, 32020) for 12–18 h. Then slices were washed with 0.1 M PB and incubated DAB (Vector Laboratories, SK-4100) for 2–10 min. After several washes in distilled water, mount the slices with Mowiol mounting medium and cover slices with a glass coverslip. The three-dimensional morphologies of neurons were reconstructed under a 100× objective and neurolucida 360 (Version 2020.3.3) software system (MicroBrightField, Vermont).

### Reporting summary

Further information on research design is available in the Nature Portfolio Reporting Summary linked to this article.

## Supplementary information

Supplementary Information

Description of Additional Supplementary Files

Supplementary Data 1

Supplementary Data 2

Supplementary Data 3

Supplementary Data 4

Supplementary Data 5

Supplementary Data 6

Supplementary Data 7

Supplementary Data 8

Reporting Summary

## Data Availability

Single-cell RNA sequencing data generated in this study have been deposited in the EMBL-EBI with the accessible links and the accession code E-MTAB-10459. Reference datasets analyzed during this study are available: *Hodge dataset*^56^ was downloaded from https://portal.brain-map.org/atlases-and-data/rnaseq. *Han dataset*^16^ was downloaded from https://db.cngb.org/nhpca/download. *Schmitz dataset*^15^ was downloaded from https://www.ncbi.nlm.nih.gov/geo/query/acc.cgi?acc=GSE169122. *Tasic dataset*^31^ was downloaded from https://portal.brain-map.org/atlases-and-data/rnaseq. *Zhu dataset*^14^ was downloaded from http://www.evolution.psychencode.org/. All data supporting the findings of this study are provided within the paper and its Supplementary Information. Source data are provided with this paper.

## Source data

Source Data

## Code availability

Computer code to process the scRNA-seq data is available as jupyter notebooks at GitHub (https://github.com/haozhaozhe/FM_V1).
