# Supplementary material for: Identification of visual cortex cell types and species differences using single-cell RNA sequencing
Source: Nat Commun. 2022 Nov 12;13:6902. doi: 10.1038/s41467-022-34590-1 (PMC9653448; doi:10.1038/s41467-022-34590-1)
Supplement: Supplementary file 1 — Supplementary Information [file 41467_2022_34590_MOESM1_ESM.pdf]

Identification of visual cortex cell types and species differences using single-cell RNA sequencing

Authors

Jia-Ru Wei[1,7], Zhao-Zhe Hao[1,7], Chuan Xu[2], Mengyao Huang[1], Lei Tang[1], Nana Xu[1], Ruifeng Liu[1], Yuhui Shen[1], Sarah A. Teichmann[2,3,*], Zhichao Miao[4,5,*], Sheng Liu[1,6,*]

Affiliations

[1]State Key Laboratory of Ophthalmology, Zhongshan Ophthalmic Center, Sun Yat-sen University, Guangdong Provincial Key Laboratory of Ophthalmology and Visual Science, Guangzhou, China.

[2]Wellcome Sanger Institute, Wellcome Genome Campus, Cambridge, UK

[3]Department of Physics, Cavendish Laboratory, University of Cambridge, Cambridge, UK

[4]GMU-GIBH Joint School of Life Sciences, Guangzhou Laboratory, Guangzhou Medical University, Guangzhou, China

[5]European Bioinformatics Institute, European Molecular Biology Laboratory, Wellcome Genome Campus, Cambridge, UK

[6]Guangdong Province Key Laboratory of Brain Function and Disease, Guangzhou, China

[7]These authors contributed equally: Jia-Ru Wei, Zhao-Zhe Hao

*Correspondence: liush87@mail.sysu.edu.cn (S.L.),
zmiao@ebi.ac.uk (Z.M.),
st9@sanger.ac.uk (S. A. T.)

*To whom correspondence should be addressed

Supplementary figures 1-11

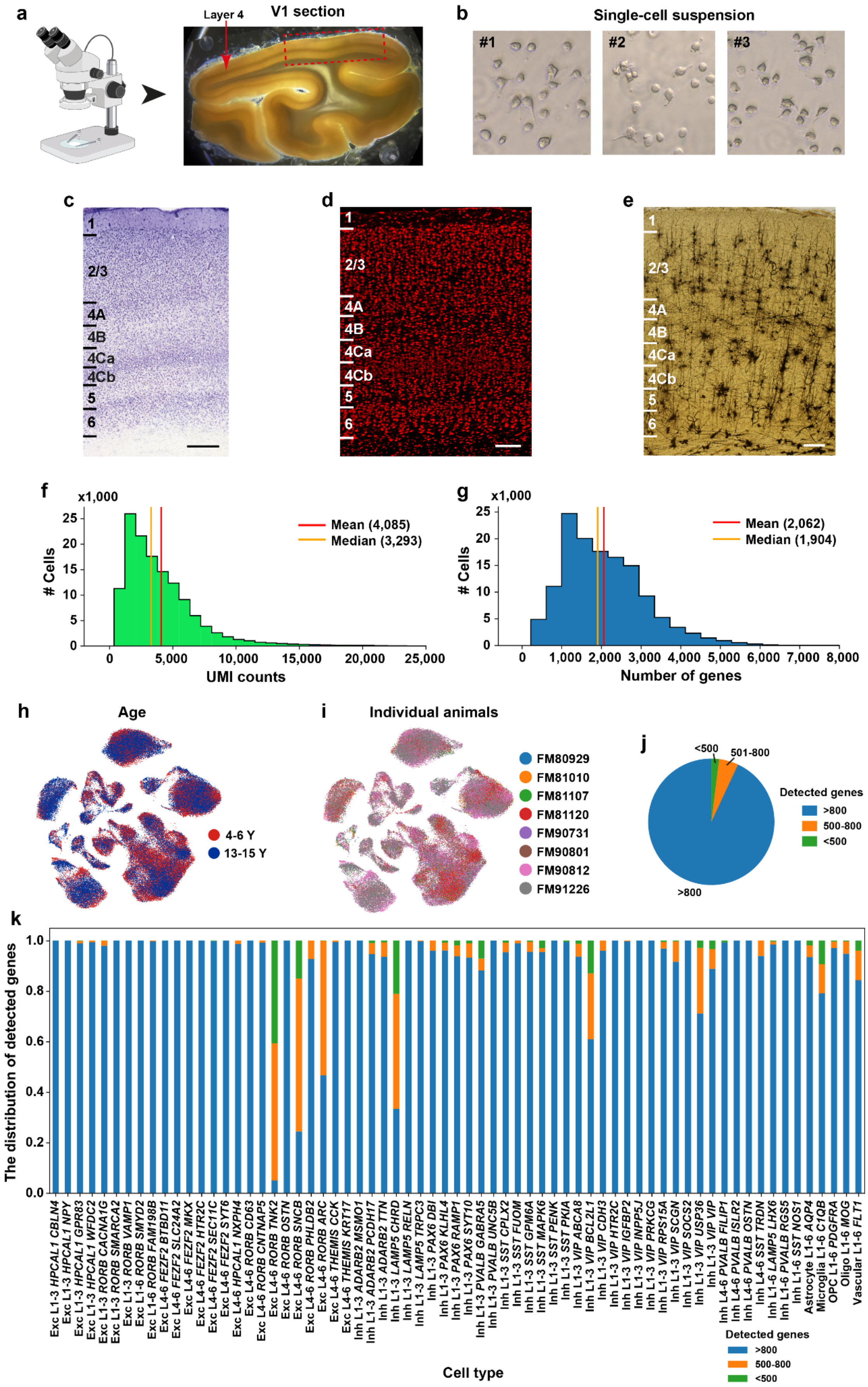

a
Layer 4
V1 section
b
Single-cell suspension
#1
#2
#3
c
1
2/3
4A
4B
4Ca
4Cb
5
6
d
1
2/3
4A
4B
4Ca
4Cb
5
6
e
1
2/3
4A
4B
4Ca
4Cb
5
6
f
x1,000
Cells
0
5
10
15
20
25
0
5,000
10,000
15,000
20,000
25,000
UMI counts
Mean (4,085)
Median (3,293)
g
x1,000
Cells
0
5
10
15
20
25
0
1,000
2,000
3,000
4,000
5,000
6,000
7,000
8,000
Number of genes
Mean (2,062)
Median (1,904)
h
Age
4-6 Y
13-15 Y
i
Individual animals
FM80929
FM81010
FM81107
FM81120
FM90731
FM90801
FM90812
FM91226
j
<500
501-800
>800
Detected genes
>800
500-800
<500
k
The distribution of detected genes
0.0
0.2
0.4
0.6
0.8
1.0
Exc L1-3 HPCAL1 CBLN4
Exc L1-3 HPCAL1 NPY
Exc L1-3 HPCAL1 GPR83
Exc L1-3 HPCAL1 WFDC2
Exc L1-3 RORB CACNA1G
Exc L1-3 RORB SMARCA2
Exc L1-3 RORB VAMP1
Exc L1-3 RORB SMYD2
Exc L1-6 RORB FAM198B
Exc L1-6 RORB BTBD11
Exc L4-6 FEZF2 SLC24A2
Exc L4-6 FEZF2 MKX
Exc L4-6 FEZF2 HTR2C
Exc L4-6 FEZF2 SEC11C
Exc L4-6 FEZF2 SYT6
Exc L4-6 HPCAL1 NXPH4
Exc L4-6 RORB CD63
Exc L4-6 RORB CNTNAP5
Exc L4-6 RORB TNK2
Exc L4-6 RORB OSTN
Exc L4-6 RORB SNCB
Exc L4-6 RORB PHLDB2
Exc L4-6 RORB ARC
Exc L4-6 THEMIS CCK
Exc L4-6 THEMIS KRT17
Inh L1-3 ADARB2 MSMO1
Inh L1-3 ADARB2 PCDH17
Inh L1-3 ADARB2 TTN
Inh L1-3 LAMP5 CHRD
Inh L1-3 LAMP5 RELN
Inh L1-3 LAMP5 TRPC3
Inh L1-3 PAX6 DBI
Inh L1-3 PAX6 KLHL4
Inh L1-3 PAX6 RAMP1
Inh L1-3 PAX6 SYT10
Inh L1-3 PVALB GABRA5
Inh L1-3 PVALB UNC5B
Inh L1-3 SST CPLX2
Inh L1-3 SST FUOM
Inh L1-3 SST GPM6A
Inh L1-3 SST MAPK6
Inh L1-3 SST PENK
Inh L1-3 SST PKIA
Inh L1-3 VIP ABCA8
Inh L1-3 VIP BCL2L1
Inh L1-3 VIP CDH3
Inh L1-3 VIP HTR2C
Inh L1-3 VIP IGFBP2
Inh L1-3 VIP INPP5J
Inh L1-3 VIP PRKCG
Inh L1-3 VIP RPS15A
Inh L1-3 VIP SCGN
Inh L1-3 VIP SOCS2
Inh L1-3 VIP USP36
Inh L1-3 VIP VIP
Inh L4-6 PVALB FILIP1
Inh L4-6 PVALB ISLR2
Inh L4-6 PVALB OSTN
Inh L4-6 SST TRDN
Inh L1-6 LAMP5 LHX6
Inh L1-6 PVALB RGS5
Inh L1-6 SST NOS1
Astrocyte L1-6 AQP4
Microglia L1-6 C1QB
OPC L1-6 PDGFRA
Oligo L1-6 MOG
Vascular L1-6 FLT1
Cell type
Detected genes
>800
500-800
<500

Supplementary Figure 1. Experimental design and quality control of scRNA-seq data.

a) Stereo microscope photographs illustrating the tissue dissection strategy. The coronal section of primary visual cortex (V1) was dissected from the adult macaque brain. The drawing of the stereo microscope was created with BioRender.com.

b) Single whole-cell isolated from adult macaque V1 regions labeled by red dashline in a. Example single-cell suspension generated from macaque V1 that exhibited diverse cellular morphologies.

c) Nissl staining section reveals the detailed laminar architecture of macaque V1. Scale bar, 150 µm. *n* = 4 macaques.

d) Fluorescent Nissl staining image shows detailed laminar architecture of macaque V1. Scale bar, 150 µm. *n* = 4 macaques.

e) Golgi staining showed morphological characterization of diversity cell type in macaque V1. Scale bars, 200 µm. *n* = 3 macaques.

f) Histogram showing the distribution of UMI counts detected across the entire scRNA-seq dataset.

g) Histogram showing the distribution of number of genes detected across the entire scRNA-seq dataset.

h) UMAP visualization of the animal age.

i) UMAP plot showing 133,454 cells by the individual animal.

j) The distribution of detected genes in each cell for the whole dataset.

k) The distribution of detected genes in each cell for each cell type. Source data are provided as a Source Data file.

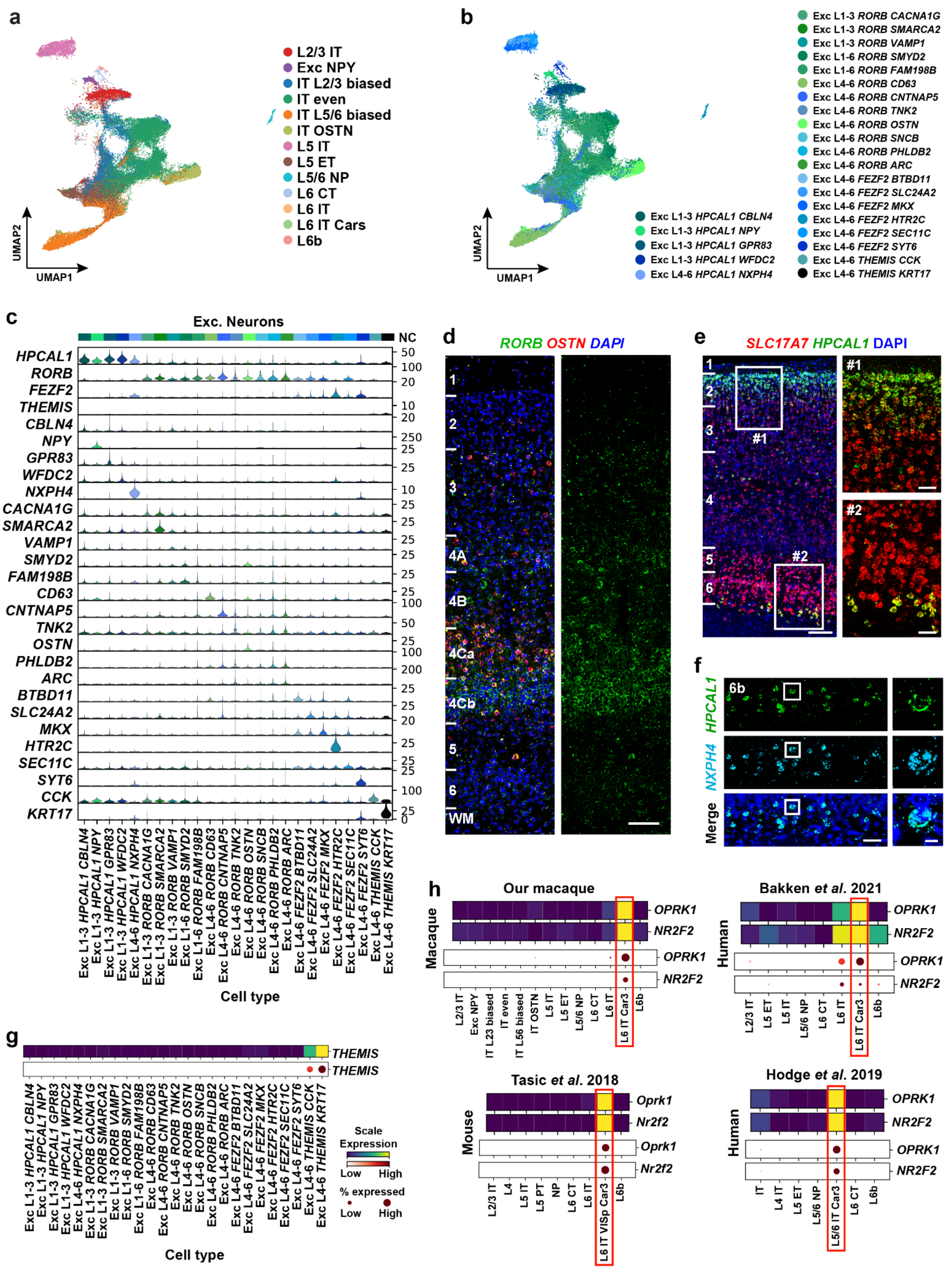
a
L2/3 IT
Exc NPY
IT L2/3 biased
IT even
IT L5/6 biased
IT OSTN
L5 IT
L5 ET
L5/6 NP
L6 CT
L6 IT
L6 IT Cars
L6b
UMAP1
UMAP2
b
Exc L1-3 RORB CACNA1G
Exc L1-3 RORB SMARCA2
Exc L1-3 RORB VAMP1
Exc L1-6 RORB SMYD2
Exc L1-6 RORB FAM198B
Exc L4-6 RORB CD63
Exc L4-6 RORB CNTNAP5
Exc L4-6 RORB TNK2
Exc L4-6 RORB OSTN
Exc L4-6 RORB SNCB
Exc L4-6 RORB PHLDB2
Exc L4-6 RORB ARC
Exc L4-6 FEZF2 BTBD11
Exc L4-6 FEZF2 SLC24A2
Exc L4-6 FEZF2 MKX
Exc L4-6 FEZF2 HTR2C
Exc L4-6 FEZF2 SEC11C
Exc L4-6 FEZF2 SYT6
Exc L4-6 THEMIS CCK
Exc L4-6 THEMIS KRT17
Exc L1-3 HPCAL1 CBLN4
Exc L1-3 HPCAL1 NPY
Exc L1-3 HPCAL1 GPR83
Exc L1-3 HPCAL1 WFDC2
Exc L4-6 HPCAL1 NXPH4
c
Exc. Neurons
NC
Cell type
d
RORB OSTN DAPI
e
SLC17A7 HPCAL1 DAPI
f
HPCAL1
NXPH4
Merge
6b
g
THEMIS
Cell type
Scale Expression
Low High
% expressed
h
Our macaque
Macaque
OPRK1
NR2F2
Bakken et al. 2021
Human
Tasic et al. 2018
Mouse
Oprk1
Nr2f2
Hodge et al. 2019
L6 IT Car3
L6 IT VISp Car3
L5/6 IT Car3

Supplementary Figure 2. Excitatory cell taxonomy and layer-specific markers in macaque V1.

a) The UMAP plot shows the classification of excitatory neurons by their layer distributions and projections.
b) The UMAP plot shows the excitatory neuron cell types and their layer distribution.
c) Violin plots visualizing the distribution of marker genes expression in excitatory neurons using the same color scheme as in Fig. 1b, e. The expression levels are normalized to the maximal normalized counts (NC, 10,000 counts per cell) of each gene.
d) RNAscope mFISH image of *RORB* (green) and *OSTN* (red) from macaque V1. Cortical layers 1-6 and white matter (WM) are labelled. The experiment was repeated three times independently with similar results. Scale bar, 100 µm. n = 3 macaques.
e) RNAscope mFISH for *SLC17A7* (glutamatergic cell marker) and *HPCAL1* showing co-expression in L2/3 and L6b. Scale bars: low magnification, 100 µm; high magnification, 25 µm. n = 3 macaques.
f) RNAscope mFISH showing the co-localization of *HPCAL1* and *NXPH4* in L6b. Scale bars, low magnification, 50 µm; high magnification, 10 µm. n = 3 macaques.
g) Dotplot shows the expression of *THEMIS* genes in glutamatergic neurons.
h) The expression pattern of IT *Car3* cells in primates and mice.

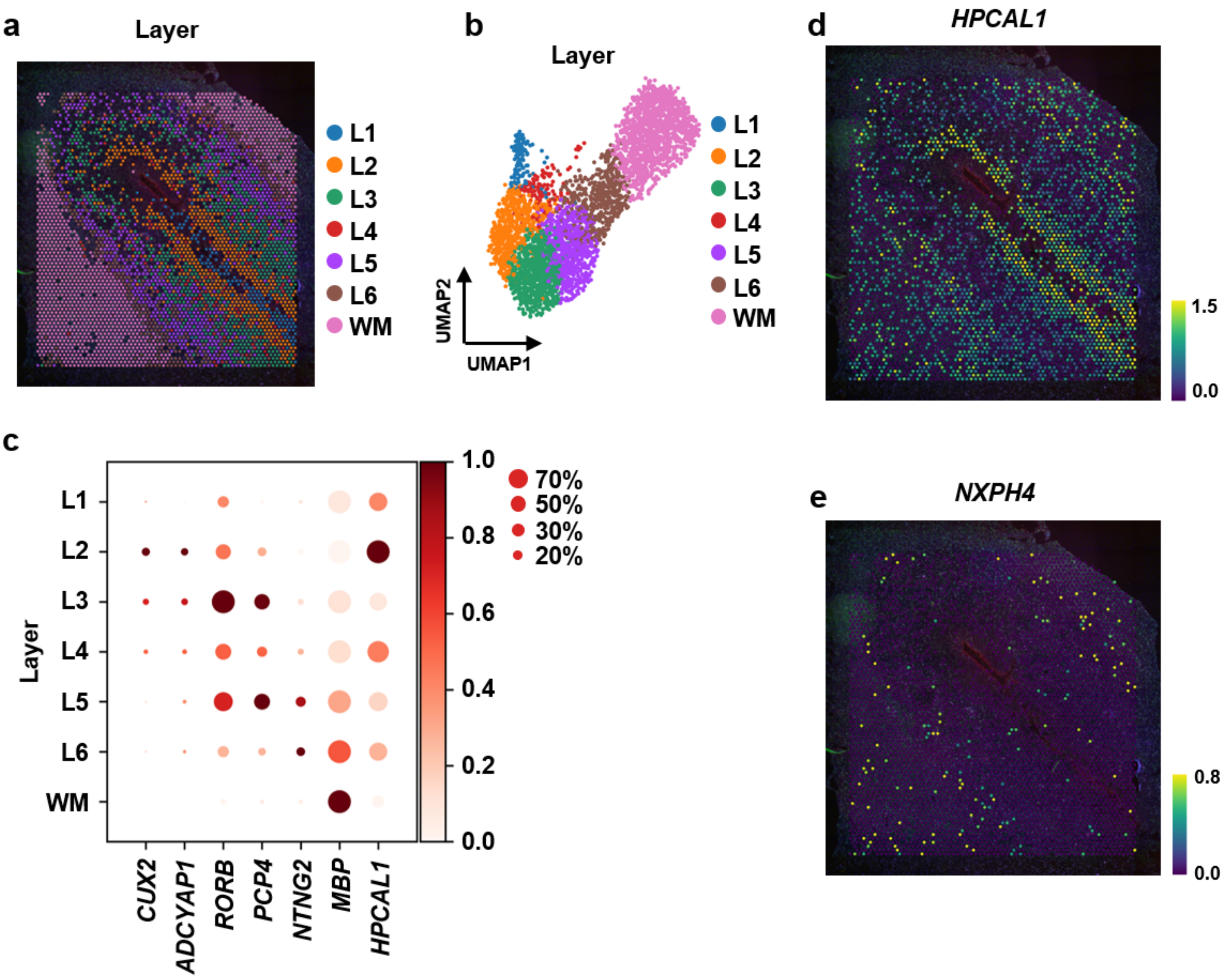
a
Layer
L1
L2
L3
L4
L5
L6
WM
b
Layer
UMAP2
UMAP1
d
HPCAL1
1.5
0.0
c
Layer
L1
L2
L3
L4
L5
L6
WM
CUX2
ADCYAP1
RORB
PCP4
NTNG2
MBP
HPCAL1
1.0
0.8
0.6
0.4
0.2
0.0
70%
50%
30%
20%
e
NXPH4
0.8
0.0

Supplementary Figure 3. ***HPCAL1*** **and** ***NXPH4*** **labels L2 and L6b of human cerebral cortex.**

a) Leiden cluster results from the dataset, annotated by layers.
b) The layer distribution of the Leiden results matching the expression pattern in a.
c) The dotplot shows the expression of genes in a.
d) *HPCAL1* gene expression is elevated in L2 and L6.
e) *NXPH4* gene expression is elevated in L6 but not in L2.

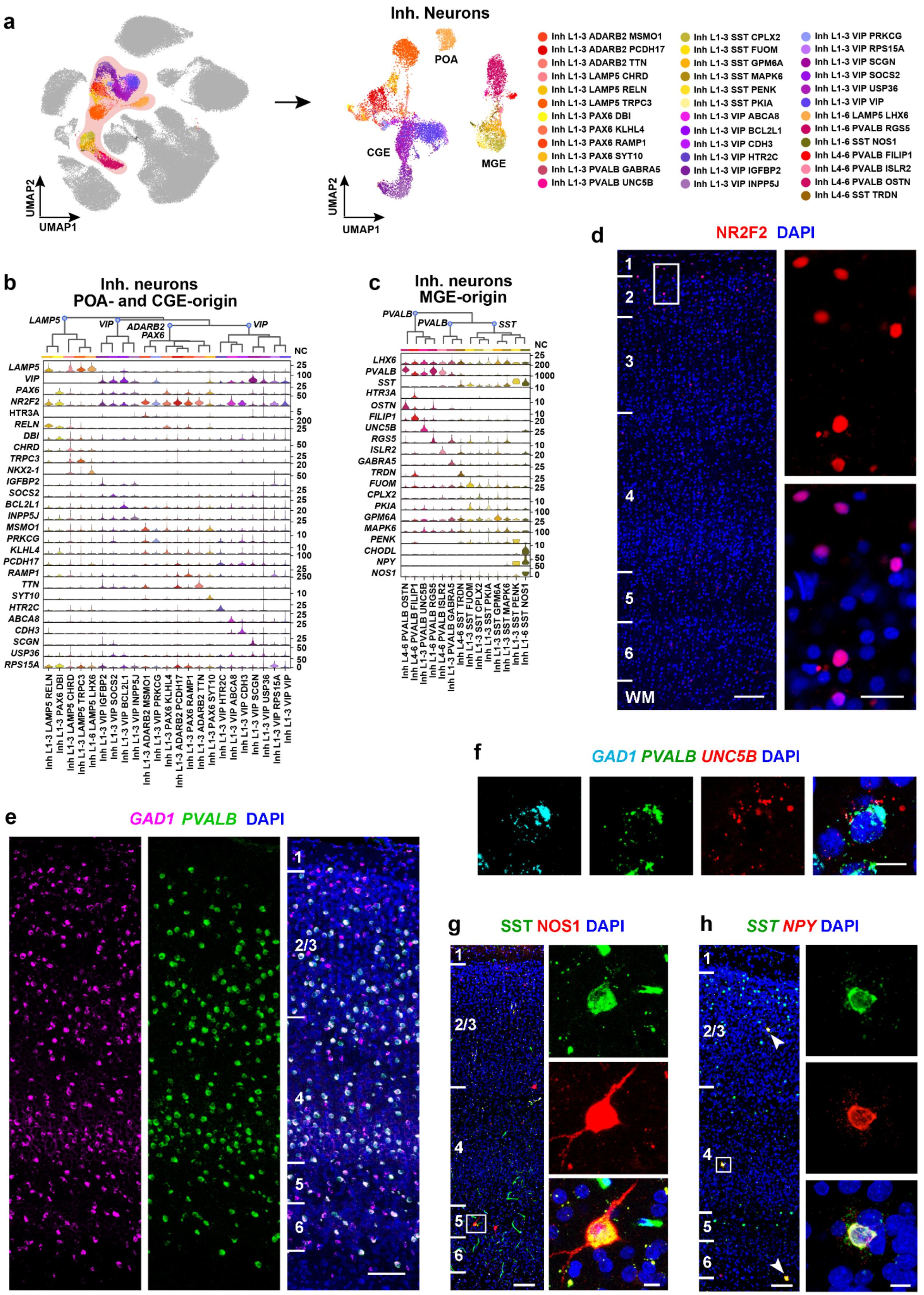
a
Inh. Neurons
UMAP2
UMAP1
POA
CGE
MGE
Inh L1-3 ADARB2 MSMO1
Inh L1-3 ADARB2 PCDH17
Inh L1-3 ADARB2 TTN
Inh L1-3 LAMP5 CHRD
Inh L1-3 LAMP5 RELN
Inh L1-3 LAMP5 TRPC3
Inh L1-3 PAX6 DBI
Inh L1-3 PAX6 KLHL4
Inh L1-3 PAX6 RAMP1
Inh L1-3 PAX6 SYT10
Inh L1-3 PVALB GABRA5
Inh L1-3 PVALB UNC5B
Inh L1-3 SST CPLX2
Inh L1-3 SST FUOM
Inh L1-3 SST GPM6A
Inh L1-3 SST MAPK6
Inh L1-3 SST PENK
Inh L1-3 SST PKIA
Inh L1-3 VIP ABCA8
Inh L1-3 VIP BCL2L1
Inh L1-3 VIP CDH3
Inh L1-3 VIP HTR2C
Inh L1-3 VIP IGFBP2
Inh L1-3 VIP INPP5J
Inh L1-3 VIP PRKCG
Inh L1-3 VIP RPS15A
Inh L1-3 VIP SCGN
Inh L1-3 VIP SOCS2
Inh L1-3 VIP USP36
Inh L1-3 VIP VIP
Inh L1-6 LAMP5 LHX6
Inh L1-6 PVALB RGS5
Inh L1-6 SST NOS1
Inh L4-6 PVALB FILIP1
Inh L4-6 PVALB ISLR2
Inh L4-6 PVALB OSTN
Inh L4-6 SST TRDN
b
Inh. neurons
POA- and CGE-origin
LAMP5
VIP
ADARB2
PAX6
VIP
NC
LAMP5
VIP
PAX6
NR2F2
HTR3A
RELN
DBI
CHRD
TRPC3
NKX2-1
IGFBP2
SOCS2
BCL2L1
INPP5J
MSMO1
PRKCG
KLHL4
PCDH17
RAMP1
TTN
SYT10
HTR2C
ABCA8
CDH3
SCGN
USP36
RPS15A
c
Inh. neurons
MGE-origin
PVALB
PVALB
SST
NC
LHX6
PVALB
SST
HTR3A
OSTN
FILIP1
UNC5B
RGS5
ISLR2
GABRA5
TRDN
FUOM
CPLX2
PKIA
GPM6A
MAPK6
PENK
CHODL
NPY
NOS1
d
NR2F2 DAPI
1
2
3
4
5
6
WM
e
GAD1 PVALB DAPI
1
2/3
4
5
6
f
GAD1 PVALB UNC5B DAPI
g
SST NOS1 DAPI
1
2/3
4
5
6
h
SST NPY DAPI
1
2/3
4
5
6

Supplementary Figure 4. Inhibitory cell taxonomy in the macaque V1.

a) Visualization of the inhibitory neuron cell types and their layer distribution. Left panel, t-SNE map using the same color scheme for inhibitory neurons and gray for all other cells. Right panel, UMAP visualises the inhibitory neurons clustered into POA-, CGE- and MGE-originated groups.
b) Violin plots of the distribution of marker gene expression of POA- and CGE-originated inhibitory neurons. The expression levels are normalized to the maximal of the cell counts that normalized to 10,000 reads per cell (NC).
c) The distribution of the marker gene expression of MGE-originated inhibitory neurons.
d) Immunostaining for NR2F2+ cells in L1 and L2. Red, NR2F2; Blue, DAPI. Scale bars, low magnification, 100 μm; high magnification, 25 μm. *n* = 3 macaques.
e) RNAscope mFISH validation showing *GAD1* (violet) and *PVALB* (green) is expressed across all V1 layers. Blue, DAPI. Scale bar, 100 μm. *n* = 3 macaques.
f) mFISH shows that chandelier cells co-expressing mRNA of *GAD1*, *PVALB* and *UNC5B*. Light blue, *GAD1*; Green, *PVALB*; Red, *UNC5B*; Blue, DAPI. Scale bar, 10 μm. *n* = 3 macaques.
g) Co-immunostaining shows the SST+ subclass SST+NOS1+ long-range projecting neurons, likely corresponding to the SST NPY and the SST CHODL cell types. Green, SST; Red, NOS1; Blue, DAPI. Scale bars, low magnification, 100 μm; high magnification, 10 μm. *n* = 4 macaques.
h) mFISH shows the *NPY* is expressed in a subset of SST+ cells, likely corresponding to SST NOS1 type and SST CHODL type. Left panel, co-labeled cells are indicated by white arrowheads and white box. Right panel, positive cell from white box in left panel. Scale bars, low magnification, 200 μm; high magnification, 10 μm. *n* = 4 macaques.

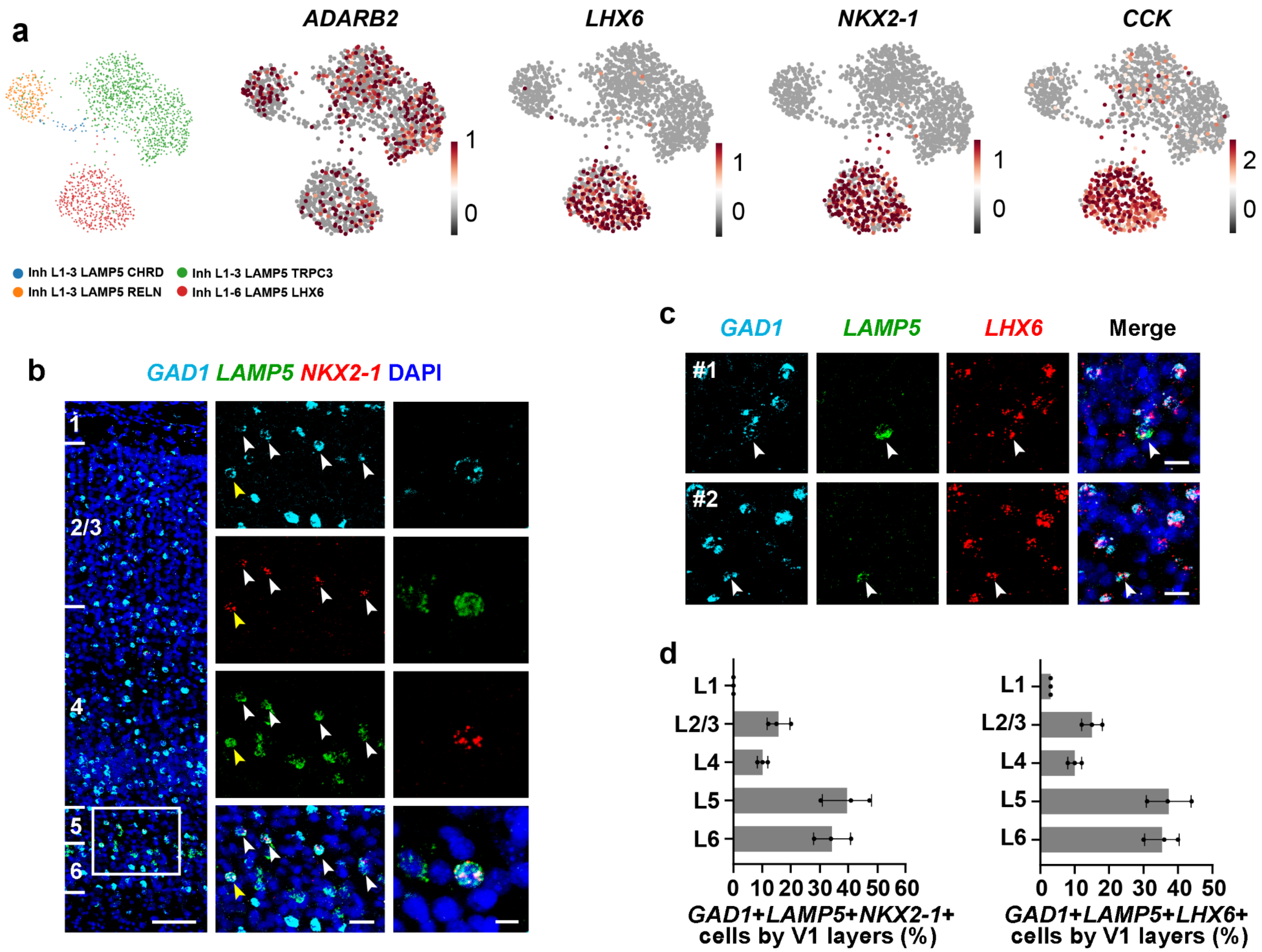
a
ADARB2
LHX6
NKX2-1
CCK
Inh L1-3 LAMP5 CHRD
Inh L1-3 LAMP5 RELN
Inh L1-3 LAMP5 TRPC3
Inh L1-6 LAMP5 LHX6
b
GAD1 LAMP5 NKX2-1 DAPI
1
2/3
4
5
6
c
GAD1
LAMP5
LHX6
Merge
#1
#2
d
L1
L2/3
L4
L5
L6
0 10 20 30 40 50 60
GAD1+LAMP5+NKX2-1+
cells by V1 layers (%)
0 10 20 30 40 50
GAD1+LAMP5+LHX6+
cells by V1 layers (%)

Supplementary Figure 5. ***LAMP5 LHX6*** **cells are the equivalent to the previously reported** ***LAMP5 LHX6*** **cell type in humans and mice.**

a) UMAPs plot of the MGE (LHX6) and CGE (ADARB2) origination markers. The LAMP5-LHX6 cell population is positive for both markers.

b) Co-expression of *GAD1*, *LAMP5* and *NKX2-1* mRNAs in macaque V1. Light blue, *GAD1*; Green, *LAMP5*; Red, *NKX2-1*; Blue, DAPI. Middle panel, positive cells that co-express all three genes are identified by yellow and white arrowheads. Right panel, a positive cell marked by yellow arrowhead in the middle panel. Scale bars, low magnification, 100 µm; middle magnification, 25 µm; high magnification, 10 µm.

c) Representative image of cells co-labeled with the *GAD1* (light blue), *LAMP5* (green) and *LHX6* (red) gene panel. Blue, DAPI. Scale bar, 10 µm. Triple-positive cells marked by white arrowheads.

d) Left panel, count of *GAD1+LAMP5+NKX2-1+* cells across layers. Data are mean ± s.d. and dots show the data points for individual macaques. Right panel, quantification of *GAD1+LAMP5+LHX6+* cells across layers. Data are mean ± s.d. and individual replicates are shown as dots. $n = 3$ macaques. Source data are provided as a Source Data file.

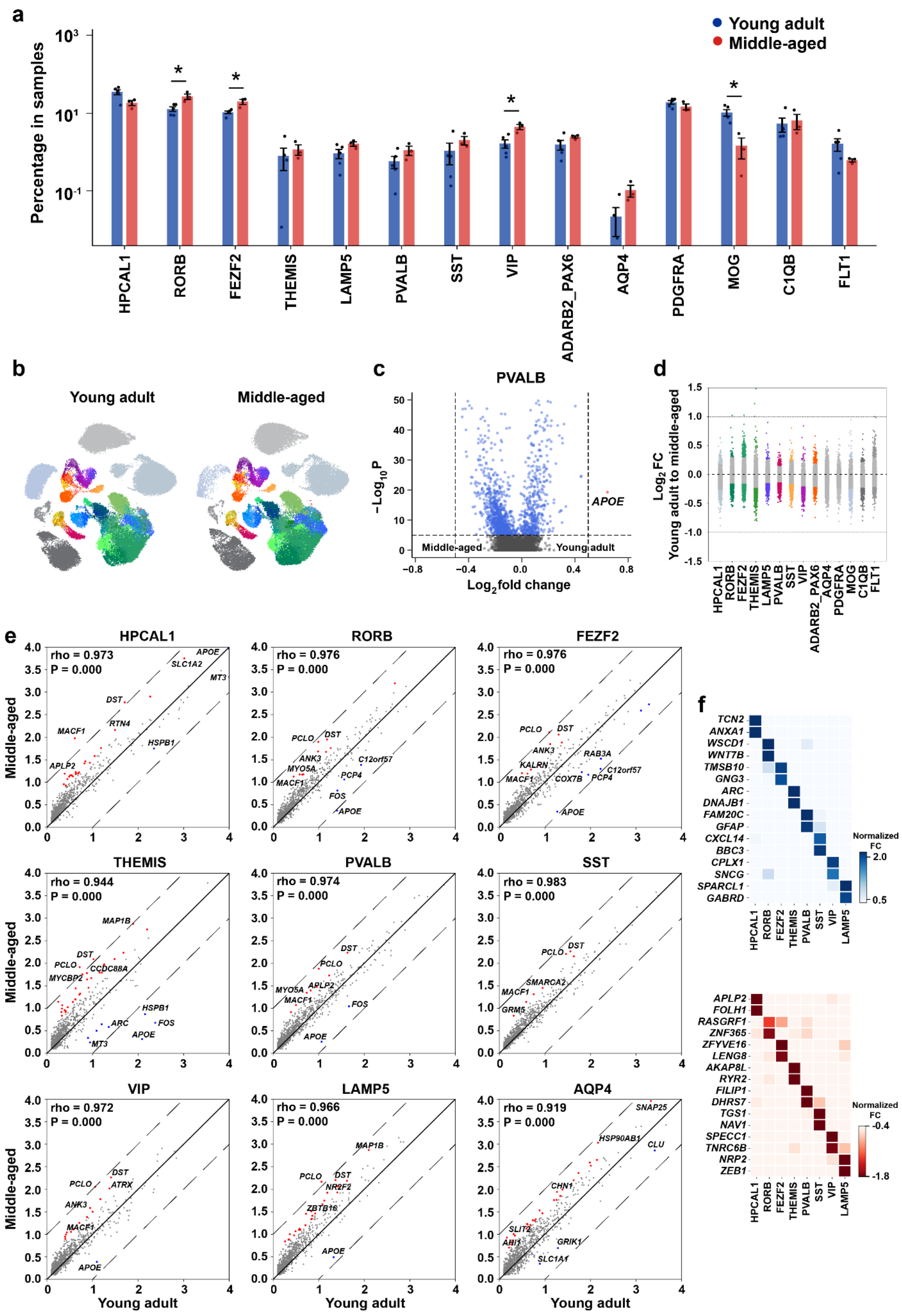
a
Young adult
Middle-aged
Percentage in samples
HPCAL1
RORB
FEZF2
THEMIS
LAMP5
PVALB
SST
VIP
ADARB2_PAX6
AQP4
PDGFRA
MOG
C1QB
FLT1
b
Young adult
Middle-aged
c
PVALB
APOE
Middle-aged
Young adult
Log2fold change
d
Log2 FC
Young adult to middle-aged
e
HPCAL1
RORB
FEZF2
THEMIS
PVALB
SST
VIP
LAMP5
AQP4
Middle-aged
Young adult
f
Normalized FC
TCN2
ANXA1
WSCD1
WNT7B
TMSB10
GNG3
ARC
DNAJB1
FAM20C
GFAP
CXCL14
BBC3
CPLX1
SNCG
SPARCL1
GABRD
APLP2
FOLH1
RASGRF1
ZNF365
ZFYVE16
LENG8
AKAP8L
RYR2
FILIP1
DHRS7
TGS1
NAV1
SPECC1
TNRC6B
NRP2
ZEB1

Supplementary Figure 6. The age-related changes in population distribution and gene expression.

a) Samples distribution grouped by age (young adult macaques, 4-6 year-old, n = 5 macaques; middle-aged macaques, 13-15 year-old, n = 3 macaques). *$P < 0.05$ by two-tailed Mann-Whitney U test. Error bars, standard error of mean. The P-value for HPCAL1 is 0.136; for RORB, 0.037; for FEZF2, 0.037; for THEMIS, 0.371; for LAMP5, 0.136; for PVALB, 0.233; for SST, 0.233; for VIP, 0.037; for ADARB2_PAX6, 0.233; for AQP4, 0.072; for PDGFRA, 0.136; for MOG, 0.037; for C1QB, 0.551; for FLT1, 0.371.
b) UMAP visualization of the 133,454 cells derived from young adult (n = 80,495 cells) and middle-aged (n = 52,959 cells) macaques V1 using the same color scheme as in Fig. 1b.
c) Volcano plot showing the differentially expressed gene of PVALB between the young and middle-aged macaques. Genes that are significantly enriched (blue dots, determined by a two-part generalized linear model implemented by MAST) and exhibit > 0.5 log2 fold-change are marked in red.
d) Strip charts showing the logarithmic-fold change (Log2 FC) of all genes between young and middle-aged macaques across all cell populations. The dashed line at the top and bottom of the plot mark the 2-fold change threshold. The dashed line in the middle indicates no differential expression. Genes that are upregulated in young adult macaques have positive FCs. Genes that are differentially expressed (Two-sided Wilcoxon rank-sum test with FDR correction for multi-comparison) and > 30% max FC are colored.
e) Pairwise comparison of gene expression between young and middle-aged macaques for selective cell populations. Red dots are genes that show > 0.5 log2 fold-change enrichment in middle-aged macaques. Blue dots are genes that show > 0.5 log2 fold change enrichment in young macaques. The solid line marks the equal expression; dashed lines indicate 2-fold enrichment. Gray dots, the mean expression of each gene of all cells of each population. All correlation rank-sum based Spearman's correlation coefficient (r) > 0.95 with $P < 0.0001$.
f) Heatmaps showing the selective genes that are upregulated in young adult macaques (upper panel) or middle-aged macaques (lower panel) exclusively for selective cell populations. Normalized FC, normalized fold-change. Source data are provided as a Source Data file.

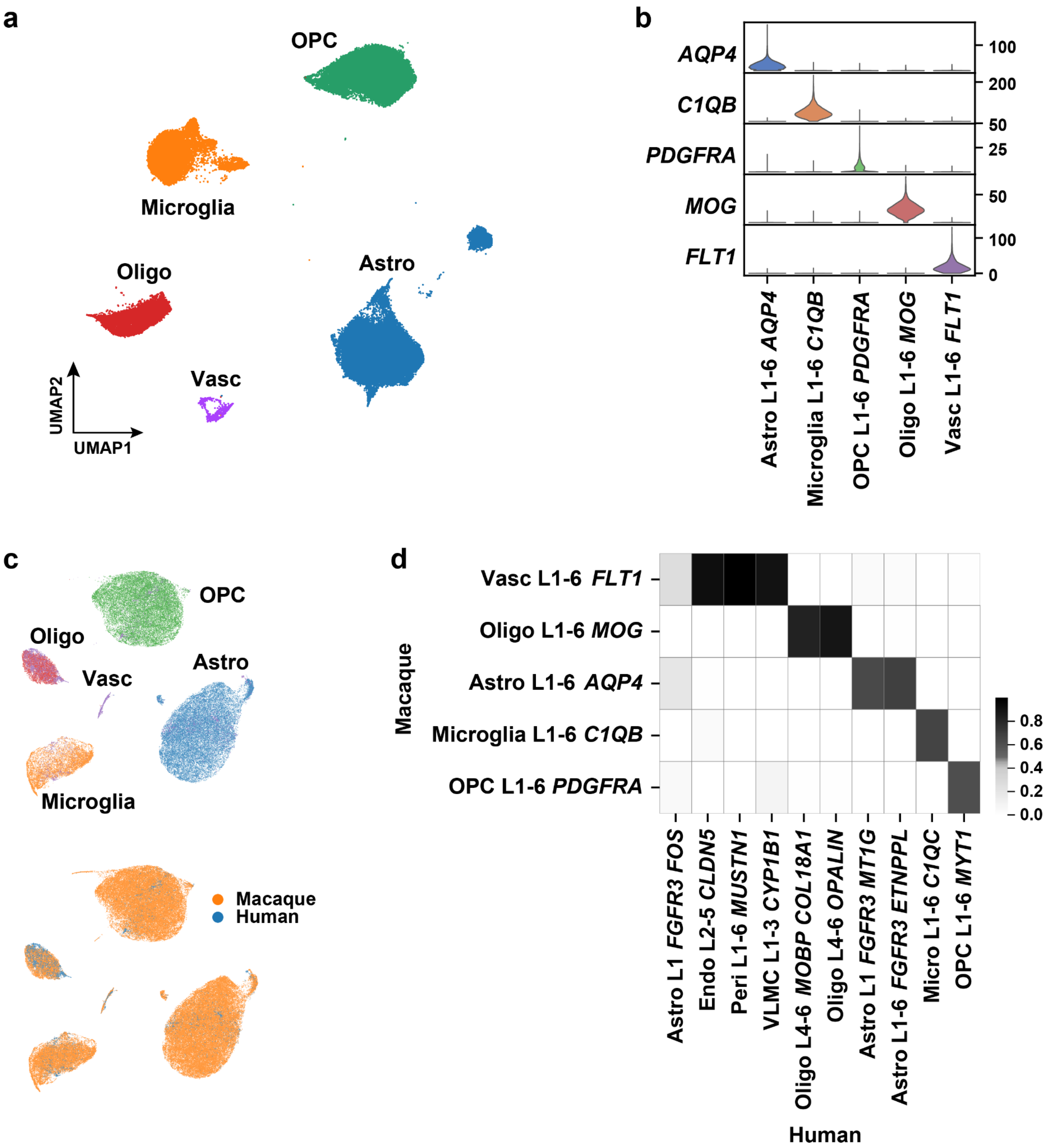
a
OPC
Microglia
Oligo
Astro
Vasc
UMAP2
UMAP1
b
AQP4
C1QB
PDGFRA
MOG
FLT1
100
200
50
25
50
100
0
Astro L1-6 AQP4
Microglia L1-6 C1QB
OPC L1-6 PDGFRA
Oligo L1-6 MOG
Vasc L1-6 FLT1
c
OPC
Oligo
Astro
Vasc
Microglia
Macaque
Human
d
Vasc L1-6 FLT1
Oligo L1-6 MOG
Astro L1-6 AQP4
Microglia L1-6 C1QB
OPC L1-6 PDGFRA
Macaque
0.8
0.6
0.4
0.2
0.0
Astro L1 FGFR3 FOS
Endo L2-5 CLDN5
Peri L1-6 MUSTN1
VLMC L1-3 CYP1B1
Oligo L4-6 MOBP COL18A1
Oligo L4-6 OPALIN
Astro L1 FGFR3 MT1G
Astro L1-6 FGFR3 ETNPPL
Micro L1-6 C1QC
OPC L1-6 MYT1
Human

Supplementary Figure 7. Aligning non-neuronal cell types between macaque and human.

a) UMAP visualization of non-neuronal cells, coloured by cell type. Astro, astrocytes; Oligo, oligodendrocytes; OPC, oligodendrocyte progenitor cells; Vasc, vascular cells.
b) The distribution of the marker gene expression of non-neuronal cell types. The expression levels are normalized to the maximal of the cell counts that normalized to 10,000 reads per cell (NC).
c) UMAP visualization of unsupervised alignment and clustering of the combination of macaque (n = 73,381 cells) and human non-neurons (n = 4,753 cells) using Harmony. Non-neuronal cells are colored by cell type (upper panel) and species (lower panel).
d) Cell-type homology of macaque and human non-neuronal cells, estimated by shared cluster membership between the two species. Endo, endothelial cells; Peri, pericytes; VLMC, vascular leptomeningeal cells; Micro, microglia.

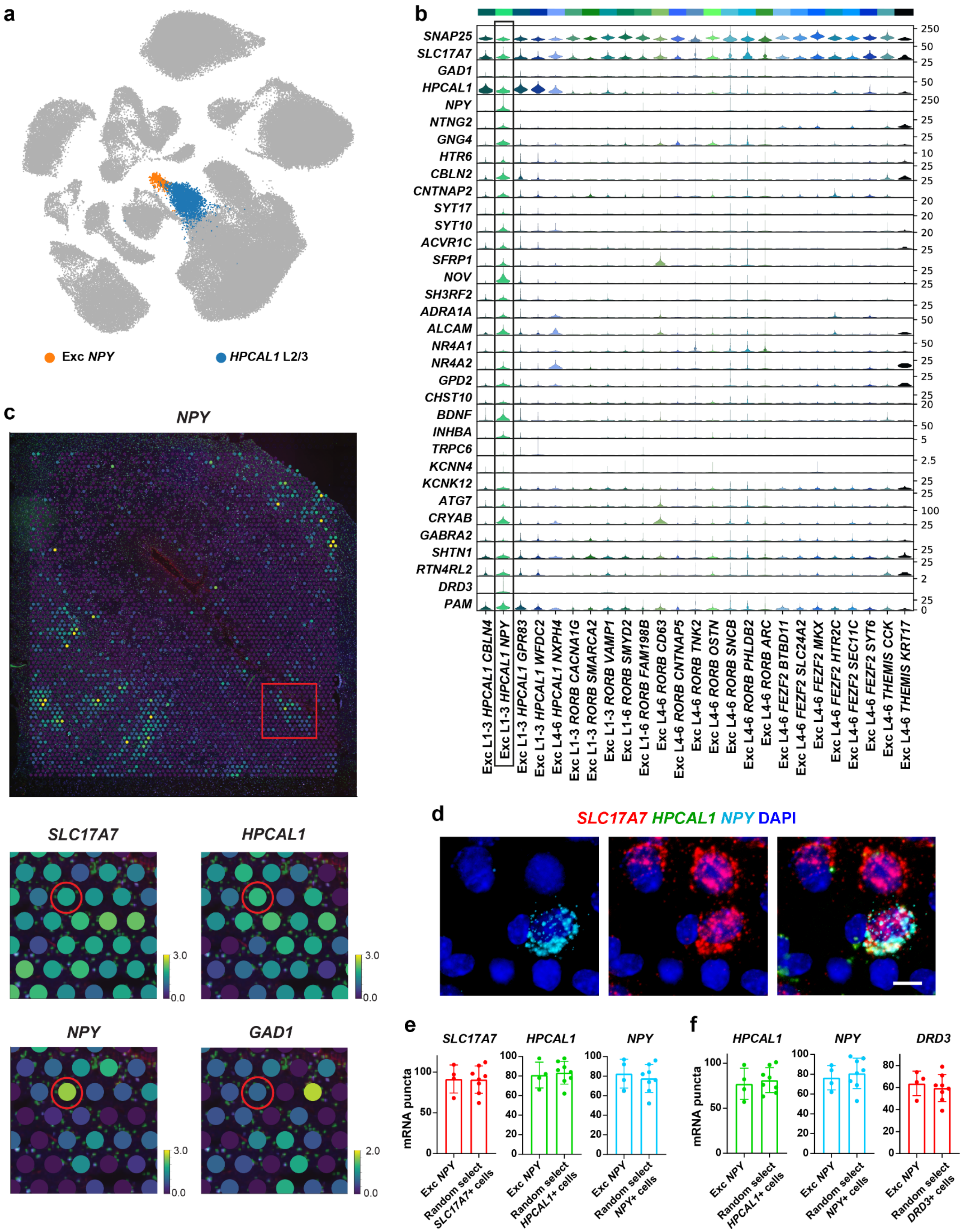

a
Exc NPY
HPCAL1 L2/3
b
SNAP25
SLC17A7
GAD1
HPCAL1
NPY
NTNG2
GNG4
HTR6
CBLN2
CNTNAP2
SYT17
SYT10
ACVR1C
SFRP1
NOV
SH3RF2
ADRA1A
ALCAM
NR4A1
NR4A2
GPD2
CHST10
BDNF
INHBA
TRPC6
KCNN4
KCNK12
ATG7
CRYAB
GABRA2
SHTN1
RTN4RL2
DRD3
PAM
Exc L1-3 HPCAL1 CBLN4
Exc L1-3 HPCAL1 NPY
Exc L1-3 HPCAL1 GPR83
Exc L1-3 HPCAL1 WFDC2
Exc L4-6 HPCAL1 NXPH4
Exc L1-3 RORB CACNA1G
Exc L1-3 RORB SMARCA2
Exc L1-3 RORB VAMP1
Exc L1-6 RORB SMYD2
Exc L1-6 RORB FAM198B
Exc L4-6 RORB CD63
Exc L4-6 RORB CNTNAP5
Exc L4-6 RORB TNK2
Exc L4-6 RORB OSTN
Exc L4-6 RORB SNCB
Exc L4-6 RORB PHLDB2
Exc L4-6 RORB ARC
Exc L4-6 FEZF2 BTBD11
Exc L4-6 FEZF2 SLC24A2
Exc L4-6 FEZF2 MKX
Exc L4-6 FEZF2 HTR2C
Exc L4-6 FEZF2 SEC11C
Exc L4-6 FEZF2 SYT6
Exc L4-6 THEMIS CCK
Exc L4-6 THEMIS KRT17
c
NPY
SLC17A7
HPCAL1
NPY
GAD1
3.0
0.0
2.0
d
SLC17A7 HPCAL1 NPY DAPI
e
SLC17A7
HPCAL1
NPY
mRNA puncta
Exc NPY
Random select SLC17A7+ cells
Random select HPCAL1+ cells
Random select NPY+ cells
f
HPCAL1
NPY
DRD3
Random select DRD3+ cells

Supplementary Figure 8. Exc *NPY* cells are a distinct cell type and may also exist in the human cortex.

a) UMAP plot visualizing the Exc *NPY* (Exc L1-3 *HPCAL1 NPY*) cell type (orange) and the *HPCAL1* L2/3 subclass (blue). Other cell subclasses are colored in gray.

b) Violin plots show the distribution of Exc *NPY* marker genes expression across all excitatory cell types. The color label at the top matches the color scheme in Fig. 1b, e. The black square indicates the *HPCAL1* L2/3 subclass, which contains only one cell type Exc *NPY* (Exc L1-3 *HPCAL1 NPY*).

c) Top panel, the expression of *NPY* of the human spatial transcriptomic dataset downloaded from the 10x Genomics database**.** Bottom panel: expression of *NPY*, *HPCAL1*, *SLC17A7* and *GAD1* genes in the wells indicated in the red box in the top panel. Notice the well in the red circle is *SLC17A7+HPCAL1+NPY+* but *GAD1-*, matching the expression pattern of Exc *NPY* cells.

d) RNAscope mFISH of another *SLC17A7+HPCAL1+NPY+* positive cell. Red, *SLC17A7*; Green, *HPCAL1*; Light blue, *NPY*; Blue, DAPI. Scale bar, 10 µm. This staining and Fig. 4c, d were captured from the V1 of 3 different macaques. *n* = 3 macaques.

e) The statistical result showing the quantification of RNAscope data represented as mean ± s.d. from three macaques. The randomly selected positive cells are in the same slice as the Exc *NPY* cells. The statistical results are not significant, as determined by two-tailed Mann-Whitney U-test. The *P*-value for *SLC17A7* is 0.933; for *HPCAL1*, 0.776; for *NPY*, 0.602. Dots represent single cells. *n* = 3 macaques.

f) Statistical analyses of RNAscope data represented as mean ± s.d. from four macaques. The randomly selected positive cells are in the same slice as the Exc *NPY* cells. The statistical results are not significant, as determined by two-tailed Mann-Whitney U-test. The *P*-value for *HPCAL1* is 0.600, for *NPY*, 0.596; for *DRD3*, 0.537. Dots represent single cells. *n* = 4 macaques. Source data are provided as a Source Data file.

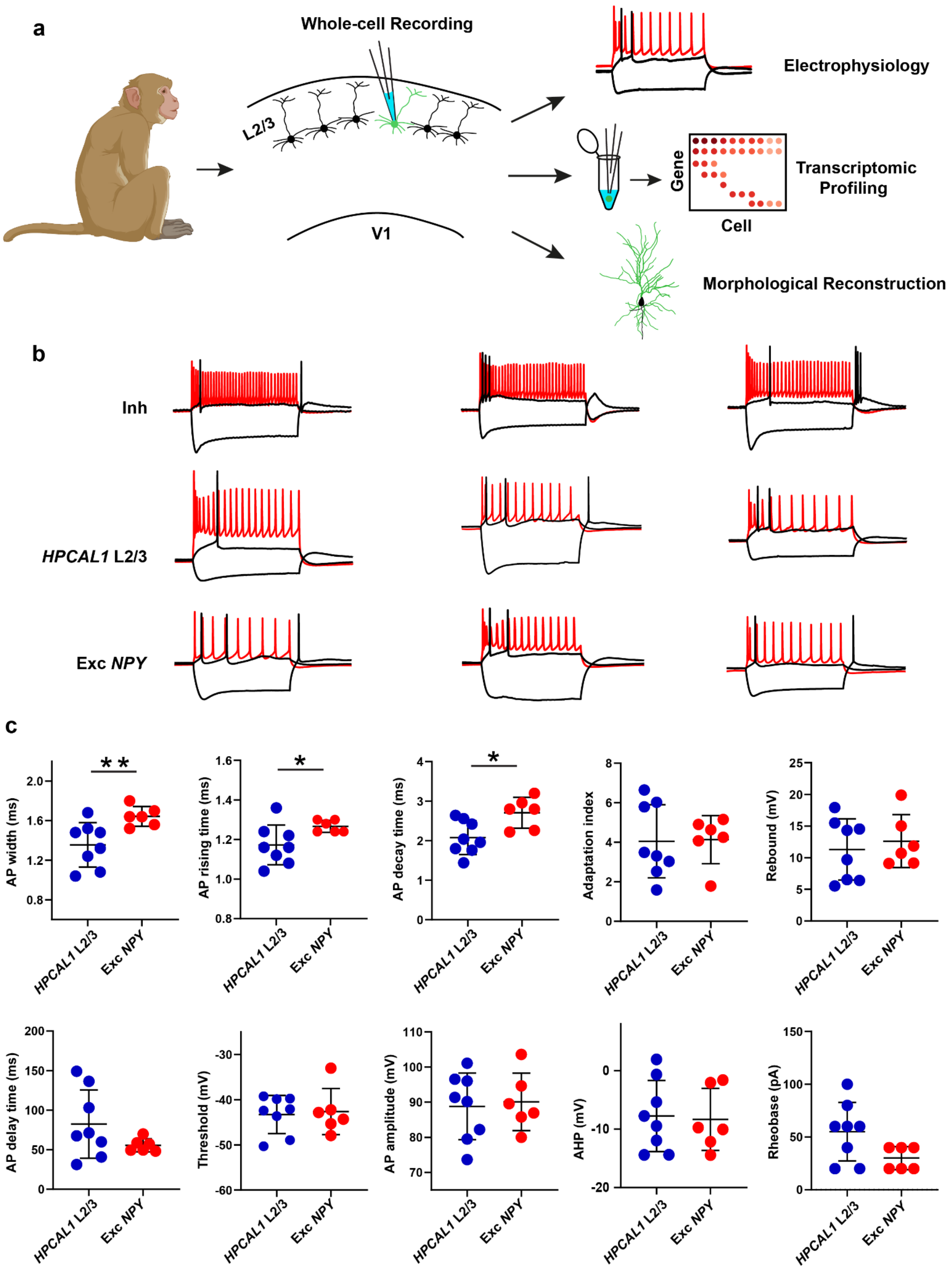
a
Whole-cell Recording
L2/3
V1
Electrophysiology
Gene
Cell
Transcriptomic Profiling
Morphological Reconstruction
b
Inh
HPCAL1 L2/3
Exc NPY
c
AP width (ms)
AP rising time (ms)
AP decay time (ms)
Adaptation index
Rebound (mV)
AP delay time (ms)
Threshold (mV)
AP amplitude (mV)
AHP (mV)
Rheobase (pA)
HPCAL1 L2/3
Exc NPY
**
*
*

Supplementary Figure 9. Patch-seq protocol and physiological data in the macaque V1.

a) Patch-seq involves the collection of morphological, physiological and transcriptomic data from the same neuron. Following electrophysiological recording and cell filling with biocytin via whole-cell patch clamp, the RNA of the cell is aspirated and processed for scRNA-seq. Drawing of macaque was created with BioRender.com.

b) Spiking responses to step currents for the three morphologically defined cell types in Fig. 4 i.

c) Plots show additional automatically extracted electrophysiological features. Circles represent single cells. Bars represent mean ± s.d.. AP, action potential; AHP, afterhyperpolarization. The significance of differences in AP width, AP rising time and AP decay time was determined using two-tailed Mann-Whitney test (**$P < 0.01$; *$P < 0.05$). The P-value for AP is 0.008; for AP rising time, 0.029; for AP decay time, 0.019; for Adaptation index, 0.852; for Rebound, 0.662; for AP delay time, 0.223; for Threshold, 0.755; for AP amplitude, 0.999; for AHP, 0.662; for Rheobase, 0.087. The $n = 8$ for *HPCAL1* L2/3; $n = 6$ for Exc *NPY*.

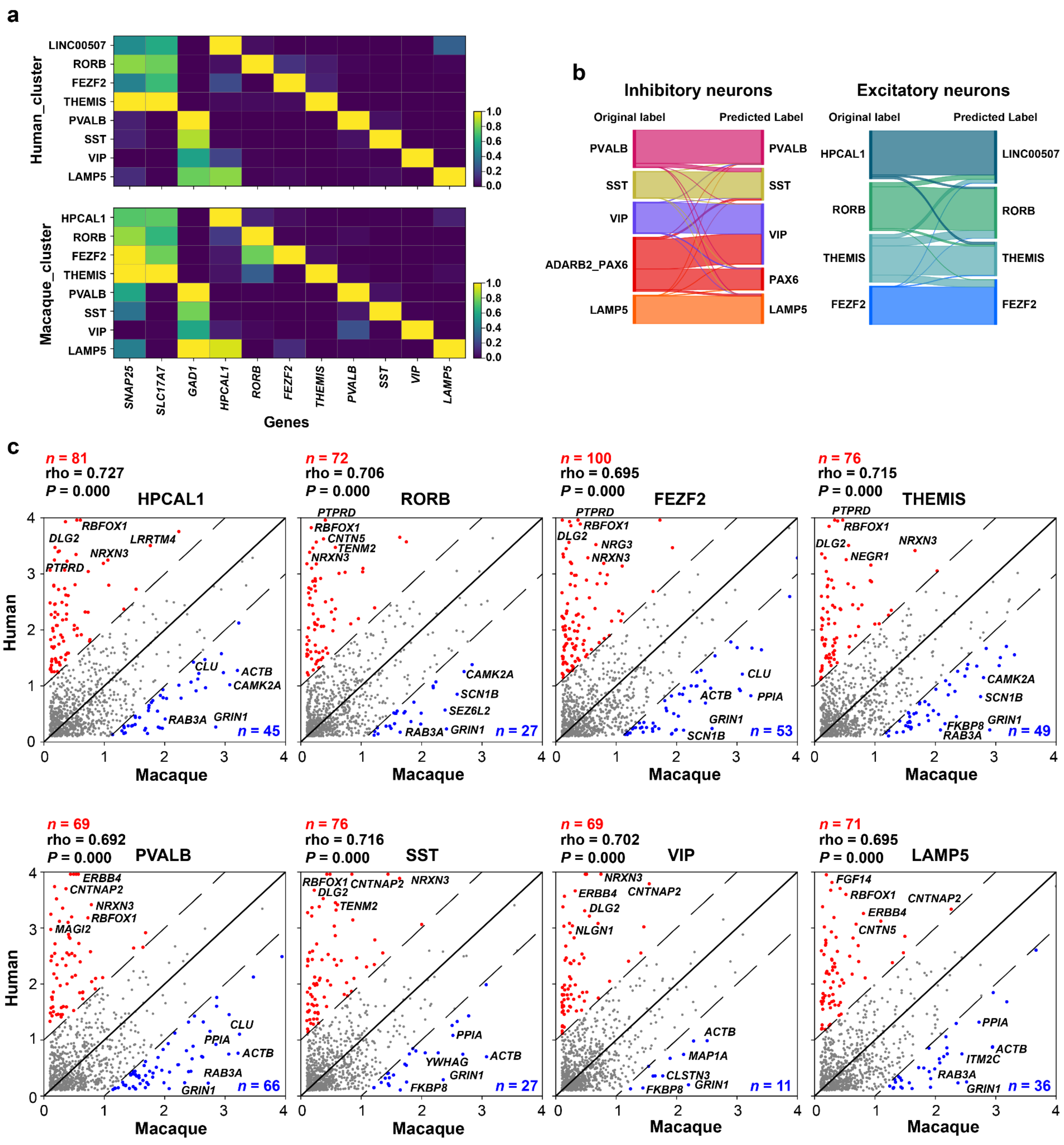
a
Human_cluster
LINC00507
RORB
FEZF2
THEMIS
PVALB
SST
VIP
LAMP5
Macaque_cluster
HPCAL1
RORB
FEZF2
THEMIS
PVALB
SST
VIP
LAMP5
1.0
0.8
0.6
0.4
0.2
0.0
SNAP25
SLC17A7
GAD1
HPCAL1
RORB
FEZF2
THEMIS
PVALB
SST
VIP
LAMP5
Genes
b
Inhibitory neurons
Original label
Predicted Label
PVALB
SST
VIP
ADARB2_PAX6
LAMP5
PVALB
SST
VIP
PAX6
LAMP5
Excitatory neurons
Original label
Predicted Label
HPCAL1
RORB
THEMIS
FEZF2
LINC00507
RORB
THEMIS
FEZF2
c
n = 81
rho = 0.727
P = 0.000
HPCAL1
RBFOX1
DLG2
LRRTM4
NRXN3
PTPRD
CLU
ACTB
CAMK2A
RAB3A
GRIN1
n = 45
n = 72
rho = 0.706
P = 0.000
RORB
PTPRD
RBFOX1
CNTN5
TENM2
NRXN3
CAMK2A
SCN1B
SEZ6L2
RAB3A
GRIN1
n = 27
n = 100
rho = 0.695
P = 0.000
FEZF2
PTPRD
RBFOX1
DLG2
NRG3
NRXN3
CLU
ACTB
PPIA
GRIN1
SCN1B
n = 53
n = 76
rho = 0.715
P = 0.000
THEMIS
PTPRD
RBFOX1
DLG2
NRXN3
NEGR1
CAMK2A
SCN1B
FKBP8
RAB3A
GRIN1
n = 49
n = 69
rho = 0.692
P = 0.000
PVALB
ERBB4
CNTNAP2
NRXN3
RBFOX1
MAGI2
CLU
PPIA
ACTB
RAB3A
GRIN1
n = 66
n = 76
rho = 0.716
P = 0.000
SST
RBFOX1
CNTNAP2
NRXN3
DLG2
TENM2
PPIA
YWHAG
ACTB
GRIN1
FKBP8
n = 27
n = 69
rho = 0.702
P = 0.000
VIP
NRXN3
ERBB4
CNTNAP2
DLG2
NLGN1
ACTB
MAP1A
CLSTN3
GRIN1
FKBP8
n = 11
n = 71
rho = 0.695
P = 0.000
LAMP5
FGF14
RBFOX1
CNTNAP2
ERBB4
CNTN5
PPIA
ACTB
ITM2C
RAB3A
GRIN1
n = 36
Human
Macaque
0
1
2
3
4

Supplementary Figure 10. Comparison of cell types and gene enrichment between human and macaque.

a) The express pattern of the canonical makers of human and macaque.

b) River plots to compare the annotation of macaque neurons with predicted labels based on human dataset [56].

c) Comparison of the gene enrichment between human and macaque for the clusters HPCAL1, RORB, FEZF2, THEMIS, PVALB, SST, VIP and LAMP5. Solid line, equal expression; dashed lines: 2-fold enrichment. Red dots, high-diversity genes that are > 2-fold enrichment in humans. Top five genes labeled by texts. Gray dots, mean expression of each neuron differentiation-related gene (n = 2,006 genes) of all cells of each population. Blue dots, high-diversity genes that are > 2-fold enrichment in macaques with the top five genes labeled by text.

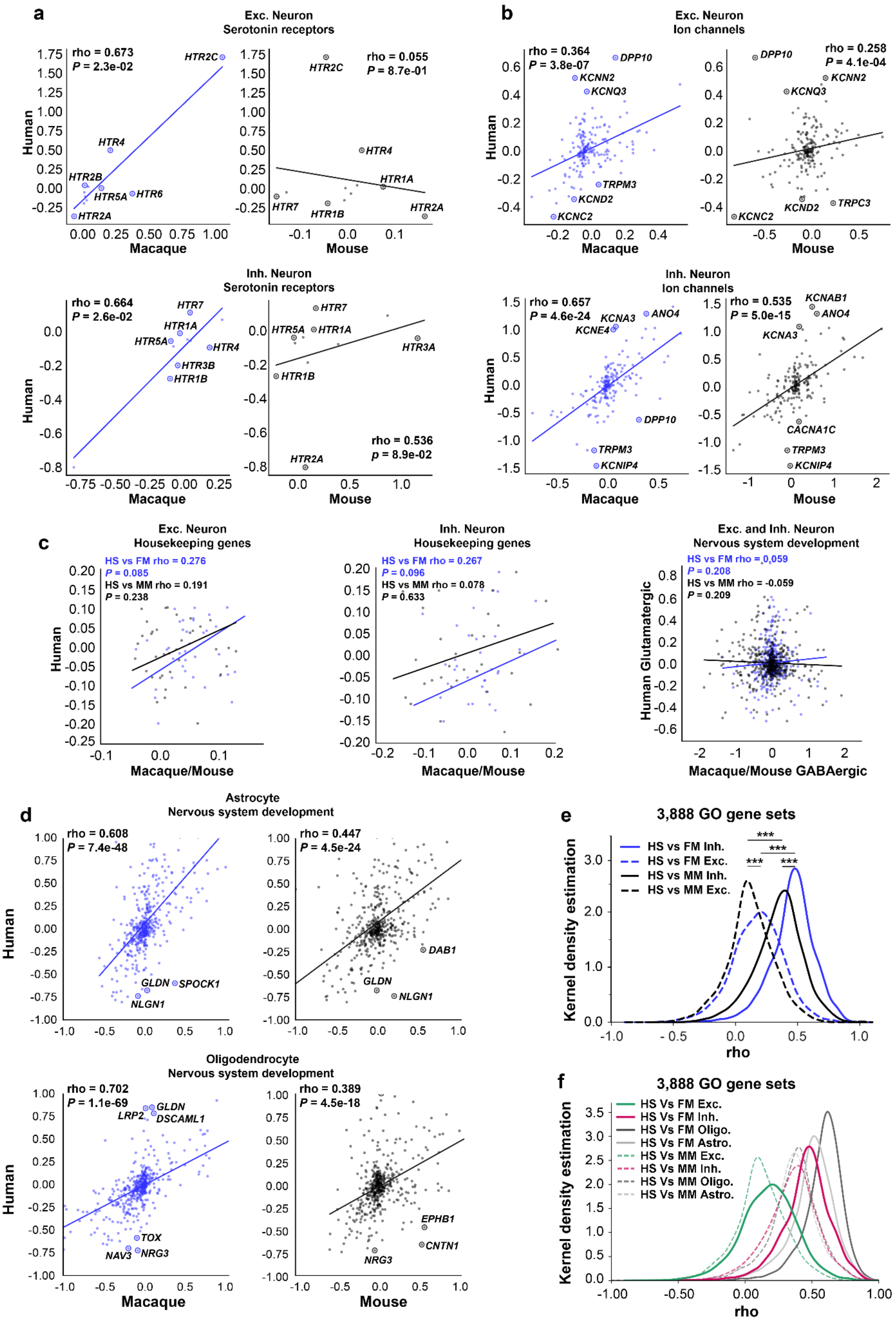
a
Exc. Neuron
Serotonin receptors
rho = 0.673
P = 2.3e-02
rho = 0.055
P = 8.7e-01
Human
Macaque
Mouse
Inh. Neuron
Serotonin receptors
rho = 0.664
P = 2.6e-02
rho = 0.536
p = 8.9e-02
b
Exc. Neuron
Ion channels
rho = 0.364
P = 3.8e-07
rho = 0.258
P = 4.1e-04
Inh. Neuron
Ion channels
rho = 0.657
P = 4.6e-24
rho = 0.535
P = 5.0e-15
c
Exc. Neuron
Housekeeping genes
HS vs FM rho = 0.276
P = 0.085
HS vs MM rho = 0.191
P = 0.238
Inh. Neuron
Housekeeping genes
HS vs FM rho = 0.267
P = 0.096
HS vs MM rho = 0.078
P = 0.633
Exc. and Inh. Neuron
Nervous system development
HS vs FM rho = 0.059
P = 0.208
HS vs MM rho = -0.059
P = 0.209
Macaque/Mouse
Human Glutamatergic
Macaque/Mouse GABAergic
d
Astrocyte
Nervous system development
rho = 0.608
P = 7.4e-48
rho = 0.447
P = 4.5e-24
Oligodendrocyte
Nervous system development
rho = 0.702
P = 1.1e-69
rho = 0.389
P = 4.5e-18
e
3,888 GO gene sets
HS vs FM Inh.
HS vs FM Exc.
HS vs MM Inh.
HS vs MM Exc.
Kernel density estimation
rho
f
3,888 GO gene sets
HS Vs FM Exc.
HS Vs FM Inh.
HS Vs FM Oligo.
HS Vs FM Astro.
HS Vs MM Exc.
HS Vs MM Inh.
HS Vs MM Oligo.
HS Vs MM Astro.

Supplementary Figure 11. The gene expression architecture is evolutionarily diverse for excitatory neurons between primates and mice.

a) The serotonin receptor-related gene expression level of human excitatory (top row) and inhibitory (bottom row) neurons are plotted against that of macaque (left, blue) and mouse (right, black), using the median z-score of each cell type within species. The Spearman's Correlation's correlation coefficient (rho) and significance were calculated and shown in each plot. The correlation is stronger between human and macaque than between human and mouse, for both excitatory neurons and inhibitory neurons.
b) The ion channel-related gene expression level of human excitatory (top row) and inhibitory (bottom row) neurons are plotted against that of macaque (left panel, blue) and mouse (right panel, black).
c) The housekeeping genes express level across species and the nervous system related-genes between excitatory and inhibitory neurons did not have significant correlation.
d) The nervous system development-related gene expression of human astrocyte (top row) and oligodendrocyte (bottom row) are plotted against that of macaque (left panel, blue) and mouse (right panel, black).
e) The distribution of Spearman's correlation coefficient (rho) between human (HS) and macaque (FM, blue lines) or mouse (MM, black lines) for both excitatory (Exc., dashlines) and inhibitory neurons (Inh., solid lines), for 3888 GO gene sets. The correlations between human and macaque are stronger compared with between human and mouse, and are stronger between inhibitory neurons than between excitatory neurons. ****$P < 0.0001$ (P-value is 10e-30) with Kruskal-wallis test followed by Dunn's test.
f) The distribution of Spearman's correlation coefficient (rho) between human (HS) and macaque (FM, solid lines) or mouse (MM, dashlines) for excitatory (Exc., green) and inhibitory neurons (Inh., red), and for oligodendrocytes (dark gray lines) and astrocytes (light gray lines) for 3888 GO gene sets. The correlations for glias (oligodendrocytes and astrocytes) are stronger compared with the neurons.
