# Supplementary material for: Identification of visual cortex cell types and species differences using single-cell RNA sequencing
Source: Nat Commun. 2022 Nov 12;13:6902. doi: 10.1038/s41467-022-34590-1 (PMC9653448; doi:10.1038/s41467-022-34590-1)
Supplement: Supplementary file 2 — Description of Additional Supplementary Files [file 41467_2022_34590_MOESM2_ESM.pdf]

Description of Additional Supplementary Files

Title: Supplementary Data 1
Description: The differential expression genes between the *HPCAL1*+ L2/3 IT and Exc *NPY* subclasses.

Title: Supplementary Data 2
Description: KEGG enrichment analysis of the Exc *NPY* up-regulated differential expression genes. *P*-value for each term was determined by Fisher's exact test implemented in g:Profiler with FDR-adjust for multiple comparisons.

Title: Supplementary Data 3
Description: Statistics of electrophysiological properties between *HPCAL1*+ L2/3 IT and Exc *NPY*. The significance of differences between *HPCAL1*+ L2/3 IT and Exc *NPY* was determined using two-sided Mann-Whitney test.

Title: Supplementary Data 4
Description: The differential expression genes between the L4 *RORB OSTN* and IT Even subclasses.

Title: Supplementary Data 5
Description: KEGG enrichment analysis of the L4 *RORB OSTN* up-regulated differential expression genes. *P*-value for each term was determined by Fisher's exact test implemented in g:Profiler with FDR-adjust for multiple comparisons.

Title: Supplementary Data 6
Description: Biological Process enrichment analysis of the L4 *RORB OSTN* up-regulated differential expression genes. *P*-value for each term was determined by Fisher's exact test implemented in g:Profiler with FDR-adjust for multiple comparisons.

Title: Supplementary Data 7
Description: KEGG enrichment analysis of the L4 *RORB OSTN* down-regulated differential expression genes. *P*-value for each term was determined by Fisher's exact test implemented in g:Profiler with FDR-adjust for multiple comparisons.

Title: Supplementary Data 8
Description: Biological Process enrichment analysis of the L4 *RORB OSTN* down-regulated differential expression genes. *P*-value for each term was determined by Fisher's exact test implemented in g:Profiler with FDR-adjust for multiple comparisons.
