# Supplementary material for: Identification of visual cortex cell types and species differences using single-cell RNA sequencing
Source: Nat Commun. 2022 Nov 12;13:6902. doi: 10.1038/s41467-022-34590-1 (PMC9653448; doi:10.1038/s41467-022-34590-1)
Supplement: Supplementary file 11 — Reporting Summary [file 41467_2022_34590_MOESM11_ESM.pdf]

nature portfolio

Corresponding author(s): Sarah A. Teichmann, Zhichao Miao, Sheng Liu

Last updated by author(s): Oct 25, 2022

Reporting Summary

Nature Portfolio wishes to improve the reproducibility of the work that we publish. This form provides structure for consistency and transparency in reporting. For further information on Nature Portfolio policies, see our Editorial Policies and the Editorial Policy Checklist.

Statistics

For all statistical analyses, confirm that the following items are present in the figure legend, table legend, main text, or Methods section.

n/a	Confirmed	
☐	☒	The exact sample size (*n*) for each experimental group/condition, given as a discrete number and unit of measurement
☐	☒	A statement on whether measurements were taken from distinct samples or whether the same sample was measured repeatedly
☐	☒	The statistical test(s) used AND whether they are one- or two-sided<br>*Only common tests should be described solely by name; describe more complex techniques in the Methods section.*
☒	☐	A description of all covariates tested
☐	☒	A description of any assumptions or corrections, such as tests of normality and adjustment for multiple comparisons
☐	☒	A full description of the statistical parameters including central tendency (e.g. means) or other basic estimates (e.g. regression coefficient) AND variation (e.g. standard deviation) or associated estimates of uncertainty (e.g. confidence intervals)
☐	☒	For null hypothesis testing, the test statistic (e.g. *F*, *t*, *r*) with confidence intervals, effect sizes, degrees of freedom and *P* value noted<br>*Give P values as exact values whenever suitable.*
☒	☐	For Bayesian analysis, information on the choice of priors and Markov chain Monte Carlo settings
☒	☐	For hierarchical and complex designs, identification of the appropriate level for tests and full reporting of outcomes
☒	☐	Estimates of effect sizes (e.g. Cohen's *d*, Pearson's *r*), indicating how they were calculated

Our web collection on statistics for biologists contains articles on many of the points above.

Software and code

Policy information about availability of computer code

Data collection	Single-cell RNA-sequencing data acquisition: Illumina Novaseq 6000 and 10x Genomics with Chromium Controller Readiness Test; electrophysiological recordings: HEKA Patch master (v2.65), MultiClamp 700B Microelectrode Amplifiers and pClamp software (v11.2); morphological reconstructions: Neurolucida 360 (v2020.3.3).
Data analysis	Single-cell RNA-sequencing data analysis using Cell Ranger (10x Genomics, v3.0.2). Scrublet (v0.2.1), Scanpy (v1.4.6), Harmony (v0.0.5), SCCAF (v0.0.10), gProfiler (v1.0.0), STAR (v2.7.10a) for primary analysis of single-cell RNA-sequencing data. Images were processed and analyzed using Zeiss ZEN software suites (v23) and Adobe Photoshop software (v21). Analysis of electrophysiology data was performed using MATLAB (v9.7). Computer code to process the scRNA-seq data is available as jupyter notebooks at GitHub (https://github.com/haozhaozhe/FM_V1).

For manuscripts utilizing custom algorithms or software that are central to the research but not yet described in published literature, software must be made available to editors and reviewers. We strongly encourage code deposition in a community repository (e.g. GitHub). See the Nature Portfolio guidelines for submitting code & software for further information.

Data

Policy information about availability of data

All manuscripts must include a data availability statement. This statement should provide the following information, where applicable:

- Accession codes, unique identifiers, or web links for publicly available datasets
- A description of any restrictions on data availability
- For clinical datasets or third party data, please ensure that the statement adheres to our policy

Single-cell RNA sequencing data generated in this study have been deposited in the EMBL-EBI with the accessible links https://www.ebi.ac.uk/biostudies/arrayexpress/studies/ and the accession code E-MTAB-10459. Reference datasets analyzed during this study are available: Hodge dataset was downloaded from https://portal.brain-map.org/atlases-and-data/rnaseq. Han dataset was downloaded from https://db.cngb.org/nhpca/download. Schmitz dataset was downloaded from https://www.ncbi.nlm.nih.gov/geo/query/acc.cgi?acc=GSE169122. Tasic dataset was downloaded from https://portal.brain-map.org/atlases-and-data/rnaseq. Zhu dataset was downloaded from http://www.evolution.psychencode.org/. All data supporting the findings of this study are provided within the paper and its Supplementary Information. Source data are provided with this paper.

Human research participants

Policy information about studies involving human research participants and Sex and Gender in Research.

Reporting on sex and gender	n/a
Population characteristics	n/a
Recruitment	n/a
Ethics oversight	n/a

Note that full information on the approval of the study protocol must also be provided in the manuscript.

Field-specific reporting

Please select the one below that is the best fit for your research. If you are not sure, read the appropriate sections before making your selection.

[x] Life sciences [ ] Behavioural & social sciences [ ] Ecological, evolutionary & environmental sciences

For a reference copy of the document with all sections, see nature.com/documents/nr-reporting-summary-flat.pdf

Life sciences study design

All studies must disclose on these points even when the disclosure is negative.

Sample size	No statistical method was used to predetermine sample sizes. For single-cell RNA-sequencing (10x Genomics), whole cells were isolated from 8 macaques; the number of animals and cells were determined to ensure the biological replicates for each cell population and the number of cells in each population met or exceed the comparable published single-cell datasets (e.g. Zhu et al. 2018, Han et al. 2022).
Data exclusions	During transcriptomic data analysis, some cells within each sample were excluded for the following reasons: 1) high mitogene percentage (>30%), and 2) low UMI count (< 200), and 3) clusters were identified as doublets and excluded if they had elevated doublet score and the combined marker gene expression profiles of more than one cell type. The criteria was determined based on the distribution of the datasets and applied equally to all samples.
Replication	Single-cell RNA-sequencing (10x Genomics) data were acquired from the 8 macaques used in the study. For single-cell RNA sequencing, all attempts at replication were successful. The in situ hybridization and immunostaining experiments were replicated with more than three animals and indicated in the figure legend. The observations were validated by previous work or other methodology including situ hybridization and immunofluoscent staining.The experiments were performed based on the availability of the animals without a set frequency.
Randomization	Randomization was not applicable for this study. There is no treatment or intervention to the samples. Therefore there is no need for randomization.
Blinding	Blinding was not applicable for this study. To avoid bias, all samples were treated equally with the same rigorous criteria.

Reporting for specific materials, systems and methods

We require information from authors about some types of materials, experimental systems and methods used in many studies. Here, indicate whether each material, system or method listed is relevant to your study. If you are not sure if a list item applies to your research, read the appropriate section before selecting a response.

Materials & experimental systems

n/a	Involved in the study
☐	☒ Antibodies
☒	☐ Eukaryotic cell lines
☒	☐ Palaeontology and archaeology
☐	☒ Animals and other organisms
☒	☐ Clinical data
☒	☐ Dual use research of concern

Methods

n/a	Involved in the study
☒	☐ ChIP-seq
☒	☐ Flow cytometry
☒	☐ MRI-based neuroimaging

Antibodies

Antibodies used

Rat anti-Somatostatin, clone YC7, Millipore, Cat# MAB354, 1:50 dilution
Rabbit nNOS (C7D7) Rabbit mAb, clone C7D7, Cell Signaling Technology, Cat# 4231S, 1:200 dilution
Mouse monoclonal anti-NR2F2, clone H7147, Abcam, Cat# ab41859, 1:200 dilution
Goat anti-Rat IgG (H+L) Alexa Fluor conjugates 488, Invitrogen, Cat# A-11006, 1:1000 dilution
Goat anti-Rabbit IgG (H+L) Alexa Fluor conjugates 594, Invitrogen, Cat# A-11037, 1:1000 dilution
Goat anti-Mouse IgG (H+L) Alexa Fluor conjugates 594, Invitrogen, Cat# A-11032, 1:1000 dilution

Validation

Well characterized commercial antibodies were used.
Somatostatin, Millipore: Validated by the vendor with immunohistochemistry in rat brain tissue and PC12 cells. The antibody is also validated in the references provided by the vendor for use in Immunohistochemistry-immunofluorescence.
DOI: 10.7554/eLife.21012. https://www.sigmaaldrich.cn/CN/zh/product/mm/mab354.

nNOS (C7D7), Cell Signaling: Validated by the vendor. IF: mouse brain. IHC: mouse brain. WB: mouse and rat brain.
The antibody is also validated in the references by immunofluorescent staining in mouse brain and iris. DOI: 10.1167/iovs.62.13.21; DOI: 10.7554/eLife.73477. https://www.cellsignal.cn/products/primary-antibodies/nnos-c7d7-rabbit-mab/4231?site-search-type=Products&N=4294956287&Ntt=4231s&fromPage=plp&_requestid=4241154.

NR2F2, Abcam: Validated by the vendor. IHC: human convoluted tubule and rat glomerular tissue. WB: Human colonic carcinoma cell line and Human embryonic kidney cell line (HEK293). https://www.abcam.cn/nr2f2-antibody-h7147-ab41859.html

Animals and other research organisms

Policy information about studies involving animals; ARRIVE guidelines recommended for reporting animal research, and Sex and Gender in Research

Laboratory animals

We used adult macaque monkeys (Macaca fascicularis) at the age of 4-15 YO.

Wild animals

The study did not involve wild animals.

Reporting on sex

There are one female and seven male macaques in 10x Genomics dataset. It is therefore challenging to explore genes with sex-related differences in our analysis.

Field-collected samples

The study did not involve samples collected from the field.

Ethics oversight

All experimental procedures were approved by the Institutional Animal Care and Use Committee (IACUC) at Zhongshan Ophthalmic Center, Sun Yat-sen University, China. This study was consistent with the Principles for the Ethical Treatment of Non-Human Primates.

Note that full information on the approval of the study protocol must also be provided in the manuscript.
